# Metabolomics provide new insights into mechanisms of *Wolbachia*-induced paternal defects in *Drosophila melanogaster*

**DOI:** 10.1371/journal.ppat.1009859

**Published:** 2021-08-12

**Authors:** Hua-Bao Zhang, Zheng Cao, Jun-Xue Qiao, Zi-Qian Zhong, Chen-Chen Pan, Chen Liu, Li-Min Zhang, Yu-Feng Wang

**Affiliations:** 1 School of Life Sciences, Hubei Key Laboratory of Genetic Regulation and Integrative Biology, Central China Normal University, Wuhan, P. R. China; 2 State Key Laboratory of Magnetic Resonance and Atomic and Molecular Physics, Innovation Academy of Precision Measurement Science and Technology, Chinese Academy of Sciences, Wuhan, P. R. China; 3 University of Chinese Academy of Sciences, Beijing, P. R. China; Pennsylvania State University, UNITED STATES

## Abstract

*Wolbachia* is a group of intracellular symbiotic bacteria that widely infect arthropods and nematodes. *Wolbachia* infection can regulate host reproduction with the most common phenotype in insects being cytoplasmic incompatibility (CI), which results in embryonic lethality when uninfected eggs fertilized with sperms from infected males. This suggests that CI-induced defects are mainly in paternal side. However, whether *Wolbachia*-induced metabolic changes play a role in the mechanism of paternal-linked defects in embryonic development is not known. In the current study, we first use untargeted metabolomics method with LC-MS to explore how *Wolbachia* infection influences the metabolite profiling of the insect hosts. The untargeted metabolomics revealed 414 potential differential metabolites between *Wolbachia-*infected and uninfected 1-day-old (1d) male flies. Most of the differential metabolites were significantly up-regulated due to *Wolbachia* infection. Thirty-four metabolic pathways such as carbohydrate, lipid and amino acid, and vitamin and cofactor metabolism were affected by *Wolbachia* infection. Then, we applied targeted metabolomics analysis with GC-MS and showed that *Wolbachia* infection resulted in an increased energy expenditure of the host by regulating glycometabolism and fatty acid catabolism, which was compensated by increased food uptake. Furthermore, overexpressing two acyl-CoA catabolism related genes, *Dbi* (coding for diazepam-binding inhibitor) or *Mcad* (coding for medium-chain acyl-CoA dehydrogenase), ubiquitously or specially in testes caused significantly decreased paternal-effect egg hatch rate. Oxidative stress and abnormal mitochondria induced by *Wolbachia* infection disrupted the formation of sperm nebenkern. These findings provide new insights into mechanisms of *Wolbachia*-induced paternal defects from metabolic phenotypes.

## Author summary

*Wolbachia* are among the most successful intracellular bacteria that can infect many arthropod species as well as filarial nematodes. *Wolbachia* can manipulate the reproduction of their insect hosts to enhance their transmission through host populations. Cytoplasmic incompatibility (CI) is the most common phenotype induced by *Wolbachia* in insect hosts, which results in embryonic mortality when *Wolbachia* infected males cross with normal (uninfected) females. This indicates that the impact of *Wolbachia* infection is mainly on paternal side. Evidence has suggested that the metabolism is strongly linked with male reproduction. We were interested in investigating whether *Wolbachia*-induced metabolic changes involve in the mechanism of paternal defects in embryogenesis. Here, using comparative metabolomics method, we identified 414 potential differential metabolites related to 34 metabolic pathways between *Wolbachia-*bearing and *Wolbachia-*free male *Drosophila melanogaster*. Then, by targeted metabolomics analysis, gene overexpression, and transmission electron microscopy (TEM) techniques, we found that in *Wolbachia* infected *D*. *melanogaster*, energy metabolism and oxidative stress were enhanced, and the development of mitochondria-derived nebenkern during spermatogenesis was impaired. These may be the main causes of paternal defects induced by *Wolbachia*.

## Introduction

*Wolbachia* are endosymbiotic bacteria that frequently infect arthropods and nematodes[1,2]. They can influence host reproduction by various strategies. Cytoplasmic incompatibility (CI) is the most common phenotype induced by *Wolbachia* in insect hosts, which causes lethality of embryos derived from the crosses between *Wolbachia* infected males and either uninfected females or females carrying a different strain of *Wolbachia*. However, when the *Wolbachia*-infected males mate with females infected with the same strain of *Wolbachia*, their offsprings develop properly[1,3,4]. CI has attracted considerable interest because it can be utilized to control agricultural pests and reduce the spread of insect-borne diseases[5–7]. The fact that *Wolbachia*-infected male insects caused no or few progenies when mated with uninfected females suggests that *Wolbachia* might induce paternal defects in embryonic development in insect hosts.

Numerous studies have suggested that *Wolbachia* infection induces modified sperms. Once these sperms fertilize uninfected eggs, the maternal histone deposition in the male pronucleus is delayed, causing improper chromosome condensation and cell division, and thus resulting in embryonic mortality[8–11]. The presence of *Wolbachia* in the female can rescue these effects on the paternal chromosomes and restore the embryonic development after fertilization[10,12,13]. These ideas have been formalized into “mod-resc” (modification/rescue) model[14], which was further interpreted by several models, such as “lock-and-key” model, “titration-restitution” model, and the “mistiming” model[15]. All the models exhibit that *Wolbachia* may deposit or remove some factors in or from host sperms. Recently, researchers have proposed that *Wolbachia* could deposit some “toxic” material in maturing sperm, such as deubiquitylase or nuclease, which can cause embryonic lethality[3,16,17]. In *Drosophila melanogaster*, the *Wolbachia* deubiquitylase CidB can bind to host nuclear import receptor, thus may impair protamine-histone exchange in sperm nucleus after fertilization, which might be the main cause of the defect in maternal histone deposition onto the paternal DNA[18]. On the other hand, sets of studies have identified host factors, including some metabolic enzymes, which could contribute to the phenotypes similar to the paternal defects induced by *Wolbachia*[19–22]. For instance, *iLvE*, coding for branched-chain-amino-acid aminotransferase which mediates branched-chain amino acid biosynthesis, exhibited the highest level of down-regulation in *Wolbachia*-infected *Laodelphax striatellus* testes versus uninfected testes. Knockdown of *iLvE* in *Wolbachia*-free males led to a significant decrease in egg hatch rate[21]. Our previous work on gene expression analyses between the *Wolbachia*-infected and uninfected 3^rd^ instar larval testes of *D*. *melanogaster* showed that significantly changed genes associated with biological process were mainly involved in host metabolism, including the upregulations of genes involved in fatty acid biosynthesis, glycolysis, and glutamate synthesis[19]. Further comparative analysis of proteins from the spermathecae and seminal receptacles of females that mated with infected or uninfected male flies indicated that the largest group of differentially expressed proteins was also highly relevant to host metabolism[20]. Collectively, *Wolbachia* infection may have a notable effect on the metabolism of insect hosts.

Previous studies have indicated the interaction of metabolism with male reproduction. For example, the testis of *Drosophila* promotes carbohydrate metabolism within the adjacent intestinal portion *via* JAK-STAT signaling. In turn, the male intestine secretes citrate to the adjacent testes thus promotes sperm maturation[23]. Furthermore, evidence also reveals that metabolic regulators of lipids, including fatty acids act as key players during *Drosophila* spermatogenesis. Mutation of *DmATPCL* encoding the human ATP citrate lyase ortholog results in defects in spindle organization, cytokinesis, and fusome assembly during meiosis of spermatocytes in *D*. *melanogaster*[24]. This mutation significantly reduced the levels of fatty acids without affecting total amount of triglycerides. Supplementation with fatty acid-medium to the mutated flies induced significant cytokinesis defects leading to reduction of the frequency of irregular spermatids, which suggested that fatty acids metabolism is closely related to spermatogenesis[24]. In mammals, intratesticular infusion of lactate into the adult cryptorchidic rat testis could apparently improve spermatogenesis, indicating that lack of energy might be a cause of defects in male germ cell development [25]. As an essential metabolite, hormones in insects play an important role in both development and reproduction[26–28]. Our previous work showed that enhancing of juvenile hormone (JH) signaling pathway in male *D*. *melanogaster* resulted in significantly decreased male fertility[29]. These suggest that the metabolism is strongly linked with male reproduction, which may contribute to *Wolbachia* induced paternal defects in their insect hosts. However, it remains unclear how, or whether metabolic changes induced by *Wolbachia* infection in *D*. *melanogaster* impact male reproduction.

In this study, we first performed untargeted metabolomics by Liquid Chromatography-Mass Spectrometry (LC-MS) to screen the potential differential metabolites and metabolic pathways between *Wolbachia-*infected and uninfected 1-day-old (1d) male flies. Subsequently, targeted metabolomics was also employed to analyze host metabolism such as carbohydrate, lipid metabolism and oxidative stress in *Drosophila* after *Wolbachia* infection. Biological assays such as gene expression and overexpression were further conducted to assay potential metabolic mechanisms of paternal defects in *Drosophila* due to *Wolbachia* infection. Our results provide new insights regarding modification of male host spermatogenesis by stimulating host metabolism after *Wolbachia* infection. Given that the effects of *Wolbachia* infection on insect hosts has broad implications in the medical and agricultural fields, our findings that *Wolbachia* can enhance the metabolism and thus induce paternal defects in embryonic development in insect hosts may have great importance in reducing transmission of human diseases and economic burden.

## Materials and methods

### Fly stocks

All flies were reared on standard cornmeal-yeast-agar medium at 25°C with a photoperiod of 12 h:12 h LD (light:dark) and under non-crowded conditions (approx. 200 eggs per 50 ml vial of media in 150 ml conical flask). The *w*Mel *Wolbachia* infected *D*. *melanogaster* (Brisbane nuclear background with introgressed *w*Mel from YW) [30], designated as *w*Mel, was kindly provided by Professor Scott O’Neill (Monash University, Australia). *Wolbachia*-uninfected Dmel T flies was subsequently generated by tetracycline treatment following established protocol [31] and confirmed to be *Wolbachia*-free by PCR using *Wolbachia* surface protein gene (*wsp*) primers (S1 Table).

UAS-*Dbi* (*w*^-^/y; pUAST-CG8627-RA; pUAST-CG8627-RA) and UAS-*Mcad* (*w*^-^/y; +; pUAST-CG12262-RA) fly lines were generated as our previously reported[29]. We generated *Dbi* and *Mcad* double overexpression line by a sequence of crossing, the detailed protocols were shown in S1A Fig. *Dbi-*RNAi (*Dbi-hp*, stock ID:64850) and *Mcad-*RNAi (*Mcad-hp*, stock ID:32436) fly lines were obtained from the Bloomington Center (Indiana University). The balancer flies were kindly provided by Professor Yongqing Zhang at Institute of Genetic and Developmental Biology, Chinese Academy of Sciences, Beijing, China. The actGal4 fly line (*w*^-^/y; actGal4/cyo; +) was from Professor Shan Jin at Hubei University, Wuhan, China. The bamGal4 flies (*w*^-^/y; bamGal4; +) were kindly provided by Professor Dahua Chen at Institute of Zoology, Chinese Academy of Sciences, Beijing, China. All of these transgenic fly lines were not infected by *Wolbachia*.

*Wolbachia*-infected bamGal4 flies were generated by crossing the *Wolbachia*-free bamGal4 males with virgin infected balancer females (*w*^-^; Cyo/Sp; Mkrs:Sb /Tm6B). The detailed crossing procedures were shown in S1B Fig.

### Flies metabolomics

For untargeted metabolomics analysis with LC-MS, 100 mg of 1d *w*Mel or Dmel T males were collected and immediately frozen with liquid nitrogen, then stored at—80°C. For metabolites extraction, the frozen flies were deposited at—20°C for 30 minutes and then thawed further in a refrigerator at 4°C. About 25 mg thawed flies were mixed with 800 μl refrigerated methanol/water (1:1, v/v) in a 2 ml EP tube. After grinding for 4 min (50 Hz), the samples were kept overnight at—20°C. Then, the samples were centrifuged (30000 g, 4°C) for 20 minutes and supernatant was collected for untargeted metabolomics analysis by LC-MS. Chromatographic separation and mass spectrometry were performed using 2777 C UPLC system (Waters, UK) and Xevo G2-XS QTOF (Waters, UK). Ten microliter sample was injected for chromatographic separationthe, and the gradient program was set as follows: 0–0.1 min, 100% mobile phase solvents A (95% H_2_O/5% acetonitrile + 0.1% formic acid); 0.1–0.6 min, 100% A-50% A (Replaced by solvents B (95% acetonitrile/5% H_2_O + 0.1% formic acid)); 0.6–5 min, 50–0% A; 5–8 min, 0% A and 8–10 min, 0% A-100% A. The fractionated samples were directly added in to the mass spectrometer, and the parameters for the MS was as the method previously described by Zhou et al.[32]. In the HPLC-QTOF-MS platform, we applied MSE mode for centroid data collection and there are only MS1 (Level 1) and MS2 (Level 2). In this paper, we analyzed all the metabolomic data at MS2 (Level 2) which have the higher reliability than those at MS1 (Level 1) in MSE mode.

For targeted fatty acid methyl ester (FAME)-based GC-MS assays, 30 frozen flies were mixed with 1 ml of methanol−chloroform (2/1, v/v) containing 5 μl internal standards (50 μM methyl ester of C17:0 and C23:0) in an EP tube. Following grinding (2 minutes) and centrifuging (20187 g, 4°C) three times for 15 minutes each time, the supernatants were combined and collected for further analysis. The methylated long chain fatty acids (LCFA) were dissolved in hexane (50 μl) and analyzed on a Shimadzu 2010 Plus GC-MS spectrometer (Shimadzu Scientific Instruments, Columbia, MD) equipped with a flame ionization detector (FID) and a DB-225 capillary GC column (10 m × 0.1 mm, 0.1 μm, Agilent Technology). Helium was used as the carrier gas and the injection volume was 1 μl. Temperature of injection port and detector was set at 230°C. The programmed column temperature was as follows: the temperature of oven was increased from 55°C to 205°C at a rate of 25°C per min, kept at 205°C for 3 min and then increased to 225°C at a rate of 10°C per min. The temperature was then kept at 225°C for further 3 min. Methylated long chain fatty acids were identified by comparing retention time with a mixture of 37 fatty acid standards. The long chain fatty acids composition was subsequently quantified by comparing integrated peak areas following normalization to the internal standards.

For short chain fatty acids (SCFA)-based GC-MS assays were performed as described by Demehri et al. [33] with minor modifications. Briefly, 30 frozen flies were mixed with 400 μl of 1N HCl and 10 μl of internal standard (2,2-dimethylbutyric acid, 1mg / ml). The samples were ground 10 times (2 minutes each time with a brief break of 1 minute), and then mixed with 400 μl of ether with centrifugation (20187 g, 4°C) for 10 min. Finally, the supernatant was collected and filtered into an autosampler vial for GC-MS measurement on the GCMS-QP2010 Plus GC-MS spectrometer (Shimadzu, Japan) equipped with a CP-FFAP CB capillary GC column (25 m × 0.32 mm, 0.3 μm, Agilent Technology). Helium was used as the carrier gas and the injection volume was 1 μl. The programmed column temperature was as follows: the temperature of injection port and detector was set at 250°C, the temperature of oven was increased from 100°C to 200°C at a rate of 10°C per min. SCFAs were identified by comparing retention time with a mixture of 9 SCFAs standards.

Seven biological replicates were performed for all LC-MS or GC-MS assays.

### Energy stores assays

The levels of glucose, trehalose, glycogen, and triglycerides (TGs) were determined using D-glucose (GOPOD-Format) (Cat. No. K-GLUC, Megazyme, Bray, Ireland), trehalose (Cat. No. E-TREH, Megazyme, Bray, Ireland), glucogen (Cat. No. BC0345, Solarbio, Beijing, China) and triglyceride (Cat. No. BC0625, Solarbio, Beijing, China) assay kits according to the manufacturer’s instructions, respectively. The 3-ml cuvettes and conventional spectrophotometer were used for measuring glucose and trehalose, while 96-well plates and a FLx 800 fluorescence reader (Bio-Tek, Winooski, USA) were used to assay glycogen and triglycerides. Thirty 1d *w*Mel or Dmel T male flies were used for each biological replicate. Three biological replicates were performed for this assay.

### Body weight loss assays

For each biological replicate, thirty newly emerged male flies were weighed with a MS105DU semi-micro balances (METTLER TOLED, Switzerland) and then introduced into an empty vial (without food and water). After fasting for 12h and 24h, the body weight loss was recorded. Three biological replicates were performed for the body weight assay.

### Feeding behaviour

For each biological replicate, thirty newly emerged male flies were introduced into the feeding apparatus with capillaries (inner diameter: 0.59 mm) containing liquid food [8% (w/v) sucrose + 8% (w/v) autolyzed yeast extract] as described in our previous work[34]. The flies were fed in this apparatus for 24 h. Initial and final heights of food in the capillary were recorded at the end of the experiment. Considering the effect of evaporation on the results, we measured the relative amount of food lost in the experimental group to that in the blank group (without flies). The food uptake of flies in each experimental group was quantified by calculating the relative height reduction of the food in the capillary to the blank group [(Initial height-Final height)_experiment_—(Initial height—Final height)_blank_]. The relative food uptake in *Wolbachia* infected group to uninfected control group was recorded. Three biological replicates were performed for this assay.

### ROS, SOD, GSH measurement

Reactive oxygen species (ROS) level was assayed according to our previous work [35]. The levels of superoxide dismutase, glutathione (GSH) were determined by using the micro SOD assay kit (Cat. No. BC0175, Solarbio, Beijing, China), micro reduced GSH assay kit (Cat. No. BC1175, Solarbio, Beijing, China) according to the manufacturer’s instructions. The 96-well plates and the FLx 800 fluorescence reader were used in this assay. Thirty 1d *w*Mel or Dmel T males flies were used for each biological replicate. Three biological replicates were performed for this experiment.

### Gene expression

Quantitative reverse transcriptase PCR (qRT-PCR) was performed to investigate the relative gene expression level. For each sample, ten 1d males or 60 pair testes (for verifying the gene expression levels specifically in testes to investigate the functions of these genes in testes) of 1d males were used to extract total RNA using Trizol (Invitrogen). RNA was reverse transcribed to generate cDNA using a High Capacity cDNA Reverse Transcription kit, EasyScript (TransGen), which included DNase to remove the DNA contamination. Specific primers for tested genes were designed based on sequences from flybase database (S1 Table). QPCR was performed using a Miniopticon system (BioRad) with a TransStart Tip Green QPCR SuperMix (TransGen). The qPCR cycling program was 95°C for 2 min, followed by 40 cycles of 95°C for 10 s, 56–60°C (depending on various primers) for 20 s and 72°C for 20 s, a melting curve was constructed from 55°C to 98°C. *rp49* (stably expressed in 1-day males and testes, S2A and S2B Fig) was chosen as the housekeeping gene, and relative expression of each gene was calibrated against *rp49* using 2^-ΔΔCT^ [ΔΔC_T_ = (CT, Target—C_T, rp49_)_experiment_—(CT, Target—C_T, rp49_)_control_][36]. For qPCR experiments, we did 3 technical replicates for each biological replicate. Three biological replicates were performed for this experiment.

### Fertility test

For each biological replicate, twelve 1d males were arranged to mate with eight 3d *Wolbachia-*free virgin (Dmel T) females for overnight. The following males were used in this experiment: actGal4(I^-^: *Wolbachia*-free)>UAS-*Dbi*, bamGal4(I^-^)>UAS-*Dbi*, actGal4(I^-^)>UAS-*Mcad*, bamGal4(I^-^)>UAS-*Mcad*, bamGal4(I^+^: *Wolbachia*-infected) >*Dbi-hp*, and bamGal4(I^+^)>*Mcad-hp*. In addition, bamGal4(I^-^)>UAS-*Mcad*; UAS-*Dbi* males were designed to mate with *w*Mel females to assay the rescue effects. actGal4(I^-^)>*w*^*-*^, bamGal4(I^-^)>*w*^*-*^, and bamGal4(I^+^)>*w*^*-*^ males were used as the corresponding control. The males were then removed and only females were maintained to lay eggs for four days. Eggs were collected and incubated at 25°C and 45–70% relative humidity for about 30 h. Egg hatch rates were determined as the proportion of hatched eggs to total eggs. Three biological replicates were performed for this test.

### Transmission electron microscopy (TEM)

Thirty pairs of testes were dissected for each sample in PBS. The testes were fixed in 2.5% glutaraldehyde (0.2 M phosphate buffer, pH 7.4) at 4°C overnight, and post-fixed in 1% OsO4 for 1 h. Then, the samples were dehydrated and embedded in Araldite (EMbed 812, China) for ultrathin sections as described by Wu et al. [37]. The samples were observed and photographed using Tecnai G2 20 TWIN 200 KV transmission electron microscope (FEI, USA) and HT-7700 80 KV transmission electron microscope (Hitachi, Japan)

### Data analysis

All values are presented as mean ± SEM. Seven biological replicates were performed for LC-MS or GC-MS assays, and three biological replicates were performed for the other tests.

The raw data of LC-MS were converted into CDF format and then imported into Progenesis QI software (version 2.0) to generate visual data, which included retention time (RT), mass-to-charge ratio (m/z) values, and peak intensity. Normalized data of LC-MS were analysed and graphed using the R language package (version 4.0.2 (2020-06-22). ape and ggplot2 were used for Partial least squares-discriminant analysis (PLS-DA); heatmap were used for cluster analysis). The freely accessible database of Kyoto Encyclopedia of Genes and Genomes (KEGG, http://www.kegg.jp) was used for identification of fragment ions and annotations of metabolic pathways. The actual spectrograms were matched to the theoretical fragmentation spectrograms to identity the potential differential metabolites, and the reference secondary database was metabolic small molecules. The significance threshold was set at *P*<0.05 and fold change >1.2 or <0.8333, and with a VIP (the variable importance of projection) value more than 1.

The other statistical analyses were performed in SPSS Statistics 25, and data were graphed by GraphPad Prism 8. Differences between means were analyzed by Student’s *t*-test. Differences were regarded as statistically significant when *P* < 0.05.

## Results

### Metabolic profile

**Fig 1:**
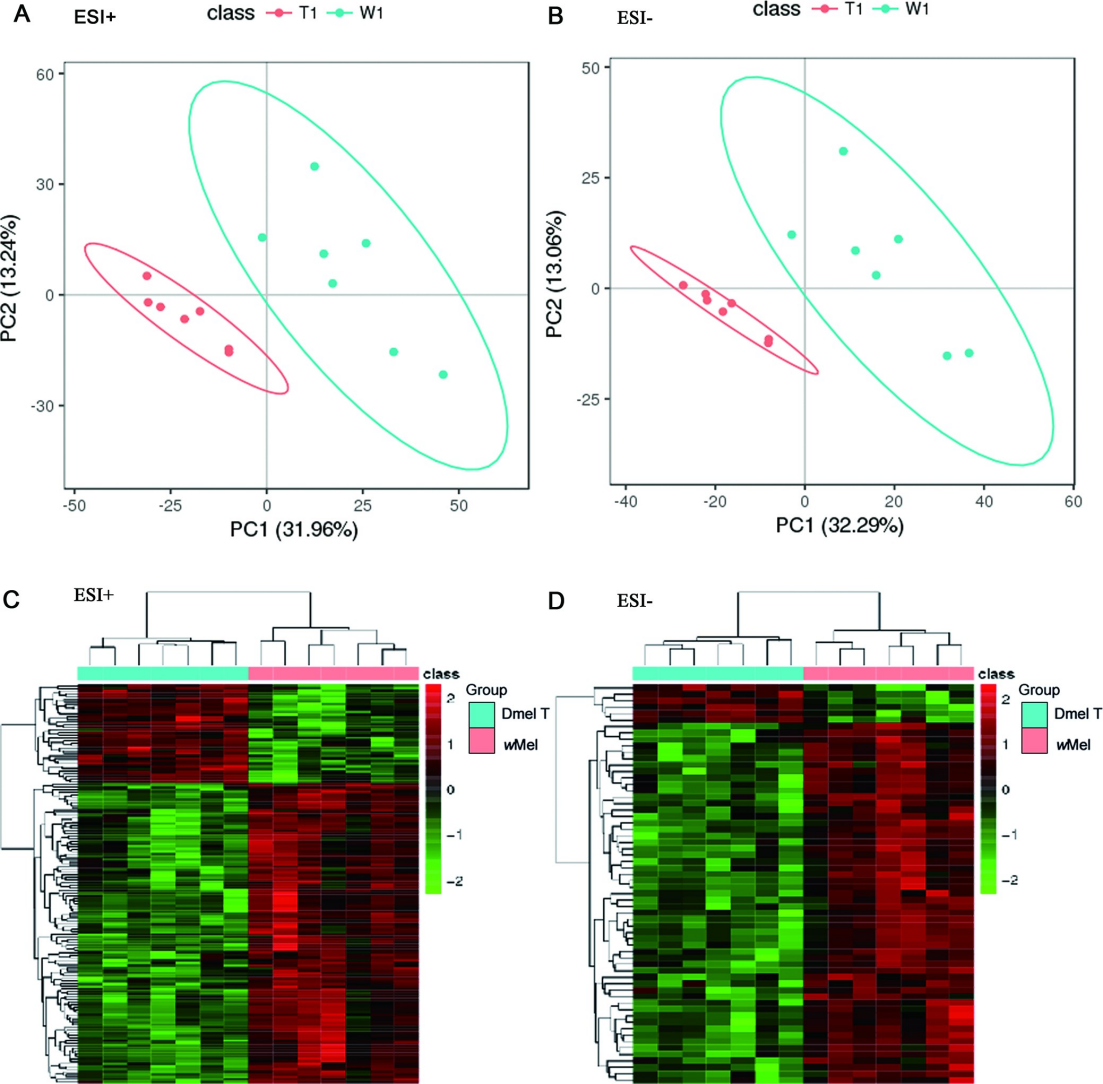
Multivariate statistical analysis of metabolomics data. Score plots of PLS-DA model in positive ion mode (A) and negative ion mode (B). Clustering analysis of differential ion in ESI+ (C) and ESI- (D). Dmel T: uninfected flies; *w*Mel: *Wolbachia*-infected flies.

A total of 9413 variables (fragments ion of metabolites) were detected by mass spectrometry [6231 peaks were detected in positive ion mode (ESI+) and 3182 peaks in negative ion mode (ESI-)]. Variables with RSD < 30% were selected for further analysis (5432 peaks in ESI+ and 2063 peaks in ESI-). Partial least squares-discriminant analysis (PLS-DA) were used to analyze the metabolomics data. The score plots for the MS signals in both positive and negative modes showed that the *Wolbachia*-infected samples were separated clearly from control samples, suggesting that the total metabolic profiling were significantly different between these two groups (Fig 1A and 1B).

The PLS-DA model displayed 216 different ions (154 in ESI+ and 62 ions in ESI-) (VIP≥1, fold-change≤0.8333 or ≥1.2, *P*<0.05) between *Wolbachia* infected and uninfected flies (S2 Table). The corresponding variable importance of projection (VIP) value can reflect the influence intensity and explanation capacity of each metabolite’s higher abundance mode effects for sample groups. Cluster analysis showed that most of different ions (172 *vs* 44) in both ESI+ and ESI-, were up-regulated by *Wolbachia* infection in *D*. *melanogaster* (Fig 1C and 1D).

### Identification of potential differential metabolites and metabolic pathways

**Fig 2:**
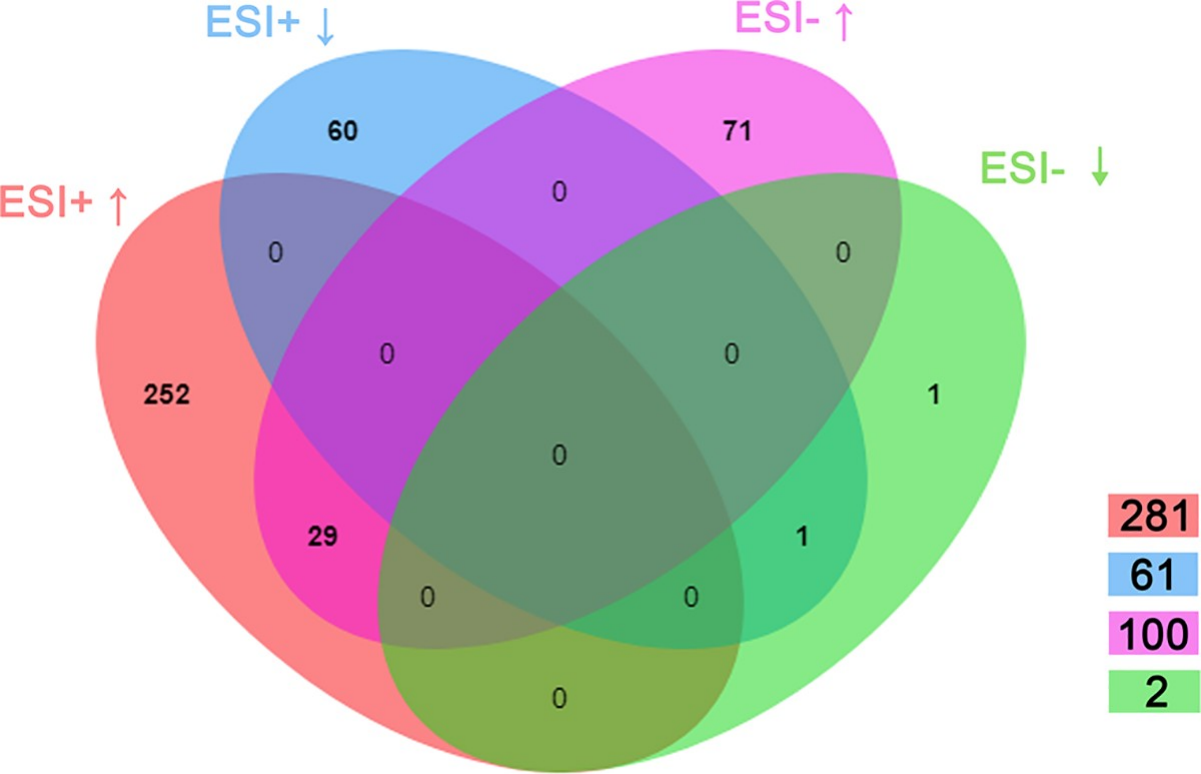
Distribution of differential metabolites in positive ion mode (ESI+) and negative ion mode (ESI-).

We next used the freely accessible database of Kyoto Encyclopedia of Genes and Genomes (KEGG, http://www.kegg.jp) to identify these different fragment ions. A total of 113 different ions were identified and 414 potential differential metabolites were correspondingly annotated (S2 Table). In positive ion mode, 281 potential metabolites were up-regulated, and the remaining 61 metabolites were down-regulated in *Wolbachia* infected flies. In negative ion mode, the quantities of up-regulated and down-regulated metabolites were 100 and 2, respectively (Fig 2). Furthermore, 29 up-regulated metabolites and 1 down-regulated metabolite due to *Wolbachia* infection appeared in both positive and negative ion modes (Fig 2).

Further annotations of metabolic pathways were made based on KEGG database. Totally, 34 metabolic pathways were found to be affected by *Wolbachia* infection (Table 1). The most influential metabolic pathway was in carbohydrate metabolism pathway, such as fructose and mannose metabolism, amino acid sugar and nucleotide sugar metabolism, glycolysis/gluconeogenesis, pentose phosphoric acid pathway, and inositol phosphate metabolism. Furthermore, metabolic pathways, including lipid metabolism, fatty acid biosynthesis, and metabolism of cofactor and vitamin membrane transport, were also affected by *Wolbachia* infection. We marked the potential metabolites in the map of metabolic pathways (Kegg ID: ko01100), and most of them are related to the carbohydrate metabolism (S3A and S3B Fig).

**Table 1:** Summary of metabolic pathways affected by *Wolbachia* infection.

Types of pathway	Pathway	Counts*	KeggID
Global and overview maps	Metabolic pathways	38	ko01100
	Biosynthesis of secondary metabolites	32	ko01110
	Microbial metabolism in diverse environments	23	ko01120
	Biosynthesis of antibiotics	36	ko01130
	Carbon metabolism	5	ko01200
	2-Oxocarboxylic acid metabolism	3	ko01210
	Fatty acid metabolism	4	ko01212
	Degradation of aromatic compounds	2	ko01220
	Biosynthesis of amino acids	2	ko01230
Carbohydrate metabolism	Glycolysis / Gluconeogenesis	5	ko00010
	Citrate cycle (TCA cycle)	1	ko00020
	Pentose phosphate pathway	4	ko00030
	Pentose and glucuronate interconversions	1	ko00040
	Fructose and mannose metabolism	12	ko00051
	Amino sugar and nucleotide sugar metabolism	9	ko00520
	Inositol phosphate metabolism	4	ko00562
	Pyruvate metabolism	2	ko00620
	Glyoxylate and dicarboxylate metabolism	1	ko00630
	Propanoate metabolism	1	ko00640
Energy metabolism	Methane metabolism	2	ko00680
Lipid metabolism	Fatty acid biosynthesis	3	ko00061
	Glycerophospholipid metabolism	2	ko00564
	Biosynthesis of unsaturated fatty acids	6	ko01040
Nucleotide metabolism	Pyrimidine metabolism	1	ko00240
Amino acid metabolism	Cysteine and methionine metabolism	1	ko00270
	Phenylalanine, tyrosine and tryptophan biosynthesis	2	ko00400
Metabolism of cofactors and vitamins	Ubiquinone and other terpenoid-quinone biosynthesis	1	ko00130
	Valine, leucine and isoleucine degradation	1	ko00280
	Thiamine metabolism	1	ko00730
	Nicotinate and nicotinamide metabolism	3	ko00760
	Pantothenate and CoA biosynthesis	3	ko00770
	Biotin metabolism	1	ko00780
	Folate biosynthesis	1	ko00790
Biosynthesis of other secondary metabolites	Monobactam biosynthesis	3	ko00261
	Streptomycin biosynthesis	5	ko00521
Membrane transport	ABC transporters	4	ko02010
Signal transduction	Two-component system	1	ko02020
Cellular community—prokaryotes	Quorum sensing	1	ko02024

*Counts refers to the number of differential metabolites matched in each metabolic pathway

### *Wolbachia* infection promotes carbohydrate and lipid metabolism of *D. melanogaster*

**Fig 3:**
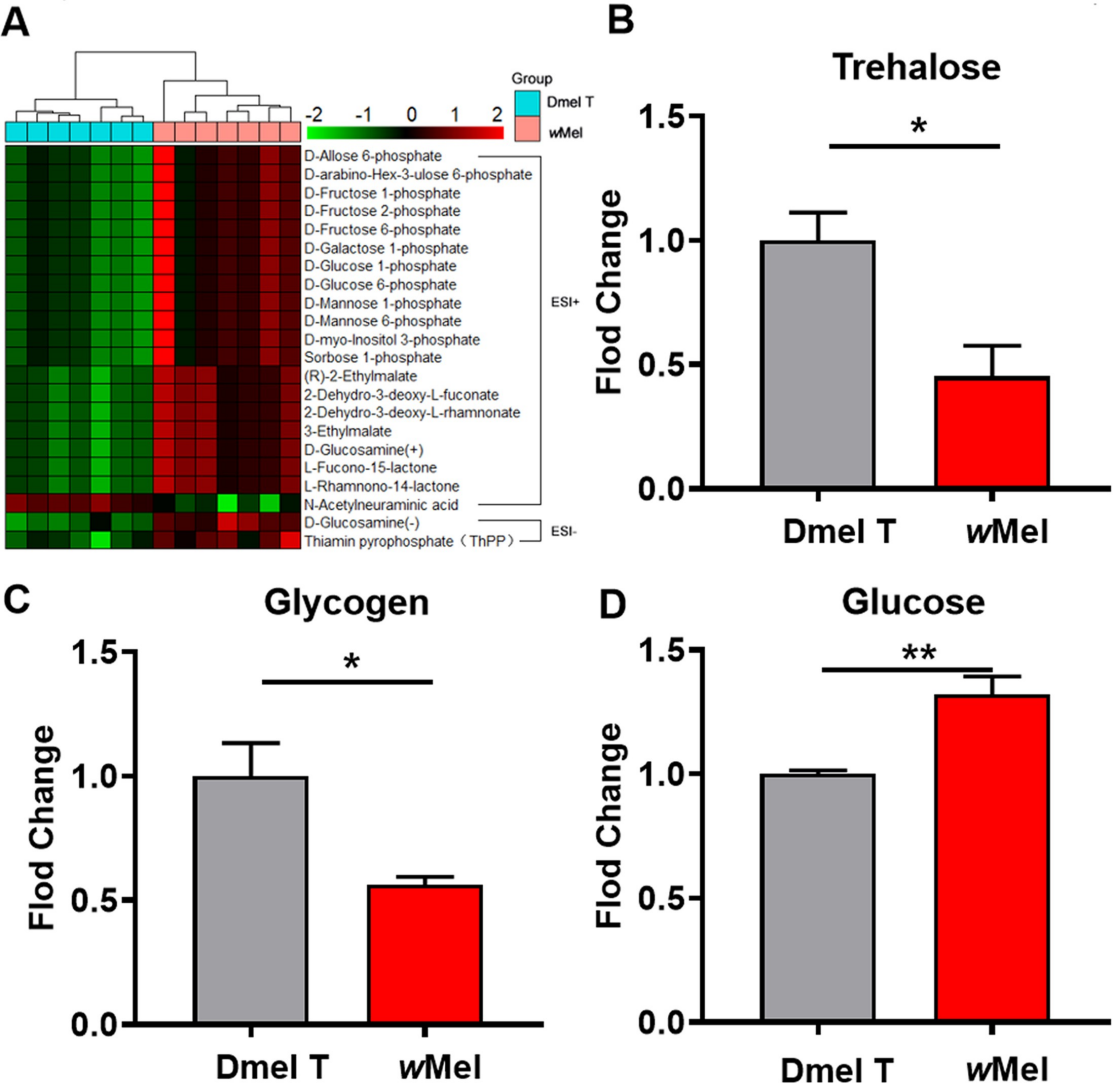
*Wolbachia* infection affected the carbohydrate metabolism of *D*. *melanogaster*. (A) Cluster analysis of differential metabolites related to carbohydrate metabolism. Corresponding measurements of trehalose (B), glycogen (C), glucose (D), in 1d male flies. Dmel T: uninfected flies; *w*Mel: *Wolbachia*-infected flies.

To better understand the effects of *Wolbachia* infection on the carbohydrate-related metabolic pathways, we applied cluster analysis to show the detailed potential metabolites and found higher levels of many glycolytic intermediates including D-glucose 6-phosphate, D-fructose 6-phosphate, and D-fructose 2-phosphate in *Wolbachia*-infected male flies compared with uninfected control flies (Fig 3A). Measurement of sugars related to major energy storage in *Drosophila* showed that the levels of both trehalose and glycogen were significantly down-regulated in the infected flies relative to uninfected ones (Fig 3B and 3C). The level of D-glucose was significantly higher in the *Wolbachia*-infected flies compared to the uninfected flies (Fig 3D). These indicate that *Wolbachia* infection may promote the degradation of sugars and boost glycolysis in male fly hosts.

**Fig 4:**
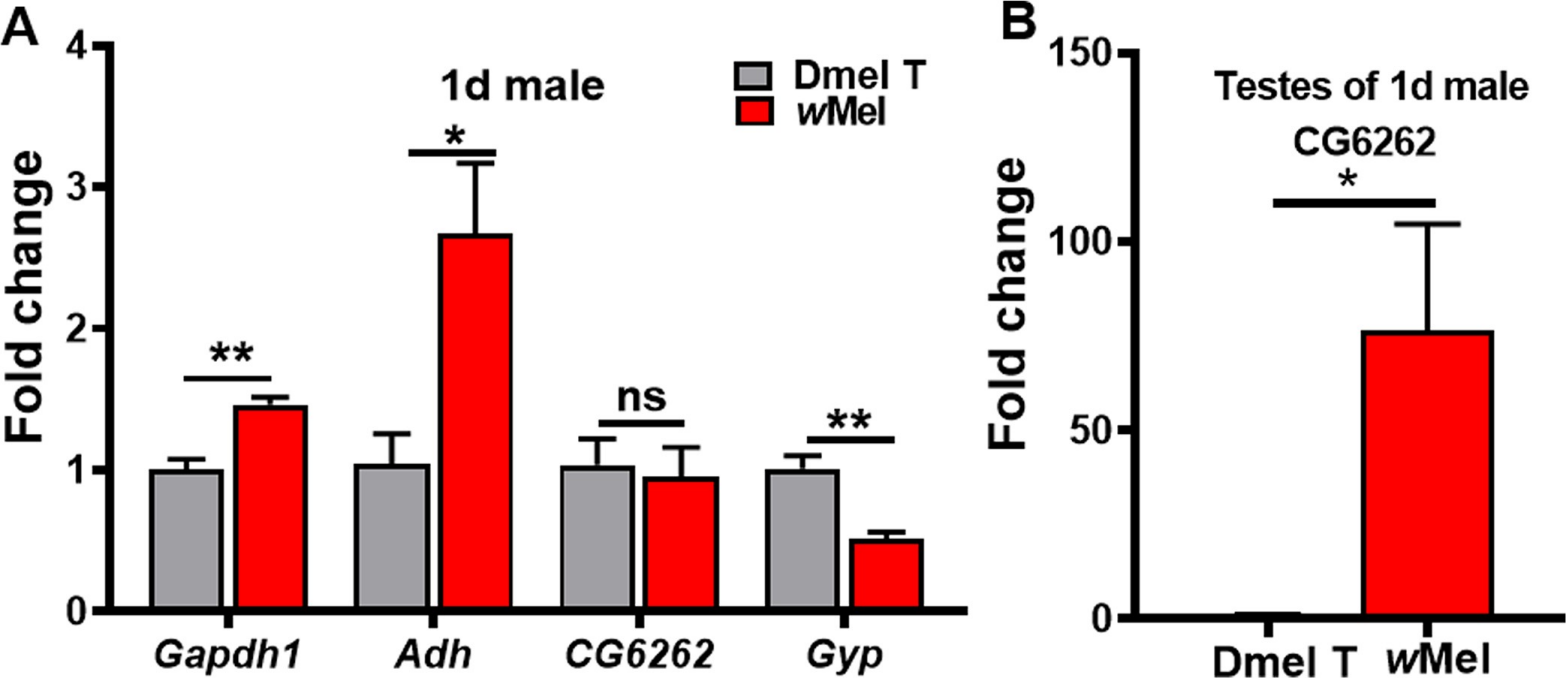
***Wolbachia* infection caused changes in mRNA levels of some glycometabolism associated genes in 1d male flies (A) and 1d male fly testes (B).** Dmel T: uninfected flies; *w*Mel: *Wolbachia*-infected flies. *P < 0.05; **P < 0.01.

We previously revealed that differentially expressed genes involved in glycolysis/gluconeogenesis, such as *Adh* (coding for alcohol dehydrogenase), *Gapdh* (coding for glyceraldehyde 3 phosphate dehydrogenase), and *CG6262* (predicted to be involved in trehalose metabolic process), were all up-regulated in male reproductive system of *Wolbachia*-infected relative to uninfected flies, while *Gyg*, coding for glycogenin glucosyl transferase, was found to be down-regulated in the presence of *Wolbachia*[19,20]. Since we used 1d whole male flies to do the comparative metabolomic analyses, to examine whether the expressions of these genes were similarly affected in whole males by *Wolbachia* infection, we applied qRT-PCR to detect the transcription level of these genes in 1d whole males. The results showed that the expression levels of *Adh* and *Gapdh* were significantly increased and the expression of *Gyg* was significantly decreased in *Drosophila* due to *Wolbachia* infection (Fig 4A). *CG6262* did not show significantly different expression in *w*Mel-infected 1d males (Fig 4A), but its mRNA level was increased by about 76-fold in testes of *Wolbachia*-infected 1d males relative to uninfected ones (Fig 4B).

**Fig 5:**
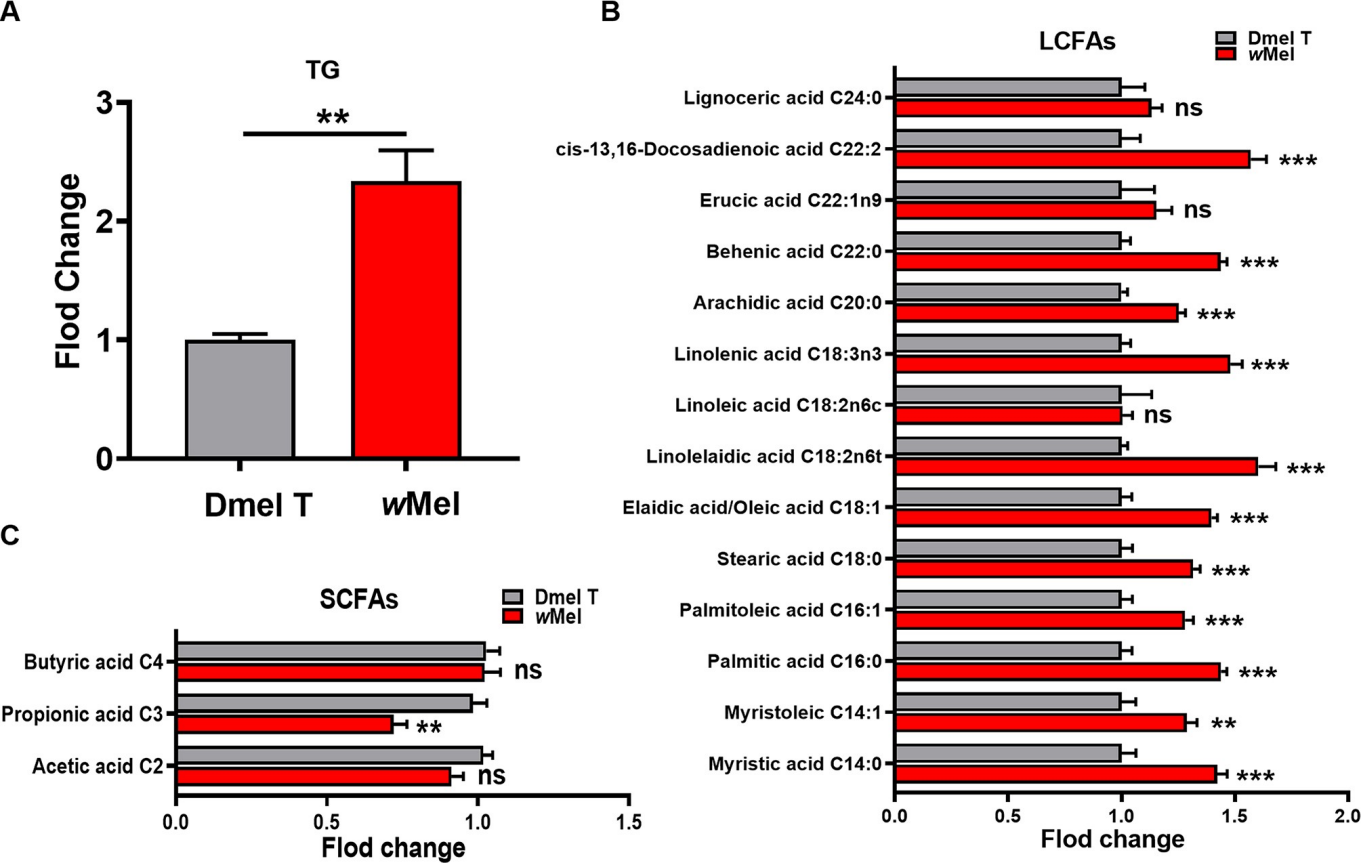
*Wolbachia* infection influenced lipid metabolism of *D*. *melanogaster*. Corresponding measurements of TG in 1d male flies (A). GC-MS analyses of long chain fatty acids (LCFAs) (B) and short chain fatty acids (SCFAs) (C) in 1d males. Dmel T: uninfected flies; *w*Mel: *Wolbachia*-infected flies. **P < 0.01; ***P < 0.001; ns: not significant.

Untargeted metabolomics analysis showed that palmitoleic acid (C16), linoleic acid (C18), linolenic acid (C18) and the corresponding acyl-CoAs were significantly elevated in *Wolbachia* infected flies (S2 Table). This implied that *Wolbachia* may affect lipid metabolism in fly hosts. *Wolbachia* infection induced significantly elevated level of triglycerides (TGs), the primary form of energy storage before oxidization in mitochondria of *Drosophila* (Fig 5A). Further targeted fatty acid compositional analysis by GC-MS showed that the levels of most LCFAs including C14, C16, C18, C20 and C22 were significantly increased in male flies due to *Wolbachia* infection (Figs 5B and S4A). GC-MS analysis of SCFAs showed that the levels of acetic acid (*P* = 0.0582) and propionic acid (*P* = 0.0015) were much lower in infected flies than those in uninfected flies (Figs 5C and S4B).

### Overexpression of *Dbi* or Mcad damaged male fertility in *D. melanogaster*

**Fig 6:**
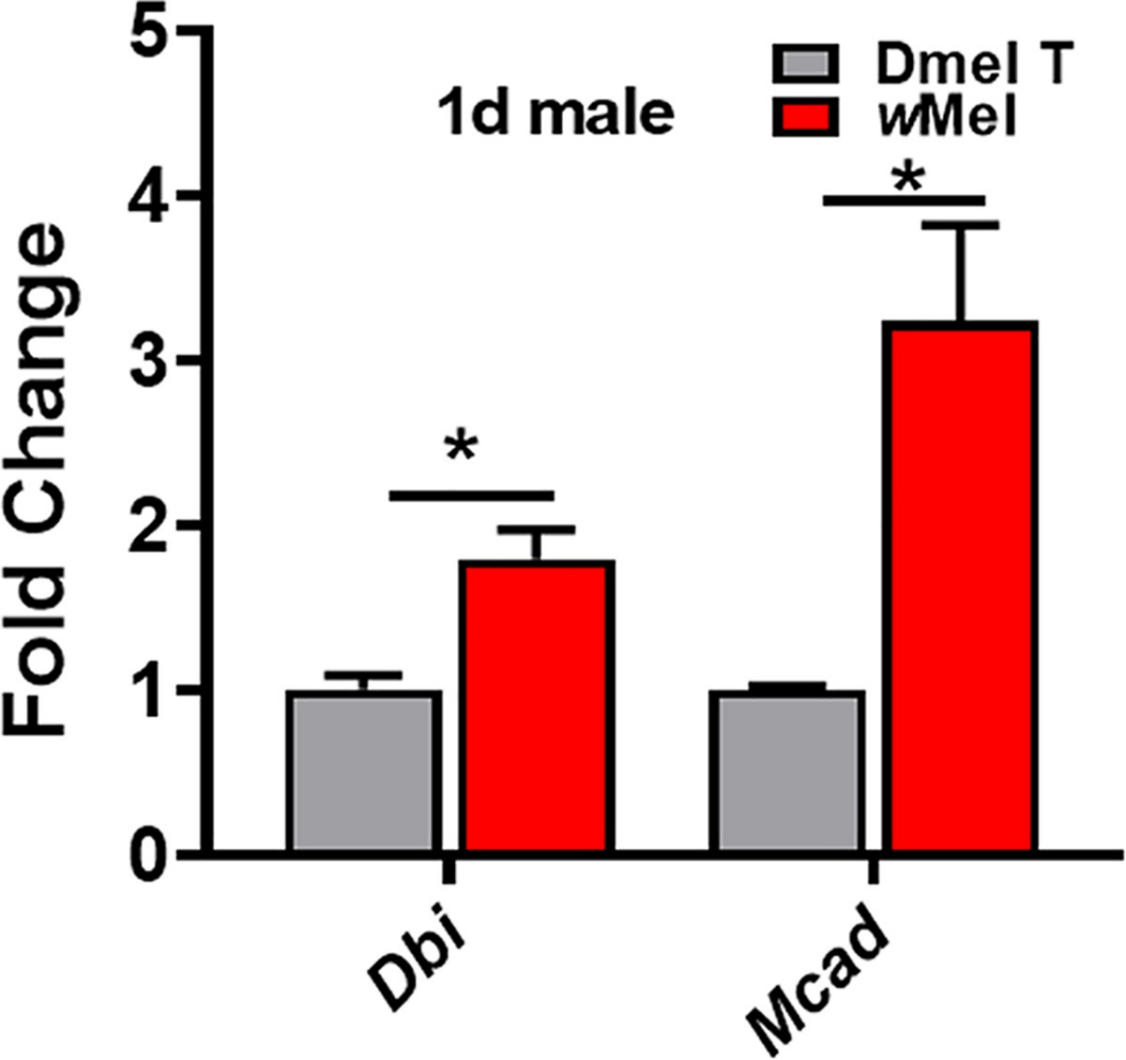
*Wolbachia* infection upregulated the transcription of *Dbi* and *Mcad*. Dmel T: uninfected flies; *w*Mel: *Wolbachia*-infected flies. *P < 0.05.

Since both untargeted and targeted metabolomics analysis showed that the lipid metabolism was enhanced in *D*. *melanogaster* in the presence of *Wolbachia*, we asked whether this was achieved by altering the activity of lipid metabolism pathways and these metabolic changes linked to the mechanism of paternal defects in embryonic development. We previously reported that *Dbi* and *Mcad* were significantly upregulated in the 3^rd^ instar larval testes of *D*. *melanogaster* due to *Wolbachia* infection[19]. The protein Dbi, also known as Acbp2 (Acyl-CoA binding protein 2), was predicted to have fatty-acyl-CoA binding activity. The mitochondrial matrix protein Mcad was reported to be able to catalyze the first reaction of mitochondrial fatty acid beta-oxidation[38]. Thus, we speculated that they may co-regulate the host fatty acid metabolism after *Wolbachia* infection. QRT-PCR analyses showed that *Wolbachia* infection significantly up-regulated the mRNA levels of *Dbi* and *Mcad* in *Drosophila* (Fig 6). Overexpressed *Dbi* (bamGal4 (I-) >*UAS-Dbi*; *UAS-Dbi*) or *Mcad* (*UAS-Mcad*) either ubiquitously or specially in testes (S5A–S5D Fig) caused a significant reduction of paternal-effect hatch rate (Tables 2 and S3), which indicated that *Wolbachia* infection may damage male fertility through up-regulating *Dbi* and *Mcad* thus enhancing lipid metabolism. We also generated flies with overexpression of both *Dbi* and *Mcad* in the testes (bamGal4(I-)>*UAS-Dbi;UAS-Mcad* (S5E and S5F Fig). In this case, only one copy of *UAS-Dbi* could be maintained) and assayed the male fertility. The result showed that the egg hatch rate was 29.43%, lower than that in *Mcad-*overexpressing group (53.17%), but higher than in the bamGal4 (I-) >*UAS-Dbi*; *UAS-Dbi* male group (2.83%) (Table 2). This suggests that, relative to *Mcad*, *Dbi* may play more important role in male reproduction, or they may play distinct, non-synergistic roles in the phenotype.

**Table 2:** Fertility test.

Cross group	Cross flies (♂ × ♀)	Egg hatch (%)	Egg counted	Comparison
1	bamGal4(I^-^)>*w*^*-*^ × Dmel T	82.05 ± 0.23	2265	
2	bamGal4 (I^-^) >*UAS-Dbi*;*UAS-Dbi* × Dmel T	2.83 ± 1.17	516	2 *vs*. 1 *P<0*.*001*
3	bamGal4(I^-^)>*UAS-Mcad* × Dmel T	53.17 ± 5.11	916	3 *vs*. 1 *P*<0.05
4	baGal4(I^-^)>*UAS-Dbi;UAS-Mcad* × Dmel T	29.43 ± 1.34	991	4 *vs*. 1 *P*<0.001
5	bamGal4(I^-^)>*UAS-Dbi;UAS-Dbi* × *w*Mel	36.91 ± 1.98	976	5 *vs*. 2 *P*<0.001
6	bamGal4(I^-^)>*UAS-Mcad* × *w*Mel	68.90± 2.98	1561	6 *vs*. 3 *P* = 0.056
7	bamGal4(I^+^)>*w*^*-*^ × Dmel T*	35.72± 1.34	897	
8	bamGal4(I^+^)>*Dbi-hp* (RNAi) × Dmel T	60.21± 1.62	809	8 *vs*. 7 *P*<0.001
9	bamGal4(I^+^)>*Mcad-hp* (RNAi) × Dmel T	50.95± 1.4	869	9 *vs*. 7 *P*<0.01

I^-^: *Wolbachia*-free; I^+^: *Wolbachia*-infected; Dmel T: tetracycline treated (*Wolbachia*-free) *Drosophila melanogaster*

*: CI cross group.

To examine the relationship of over-expression of *Dbi* and *Mcad* with *Wolbachia*-induced CI, we used *Wolbachia*-infected females to cross with *Dbi* or *Mcad* overexpressing males and found that *Wolbachia*-infected females could partially rescue the embryonic lethality defects caused by over-expression of *Dbi* or *Mcad* in fly testes, since the egg hatch rates were higher than the corresponding Dmel T female groups (Table 2). We also knocked down *Dbi* or *Mcad* in the testes of *Wolbachia* infected flies (S5G and S5H Fig), and found either *Dbi* or *Mcad* knockdown could rescue the CI phenotype caused by *Wolbachia*. When they mated with Dmel T females, the egg hatch rates were significantly higher than the corresponding control group (CI cross group) (Table 2). These results suggest that *Dbi* and *Mcad* might be partially involved in *Wolbachia*-induced CI.

### *Wolbachia* infection increased the energy expenditure and food uptake

**Fig 7:**
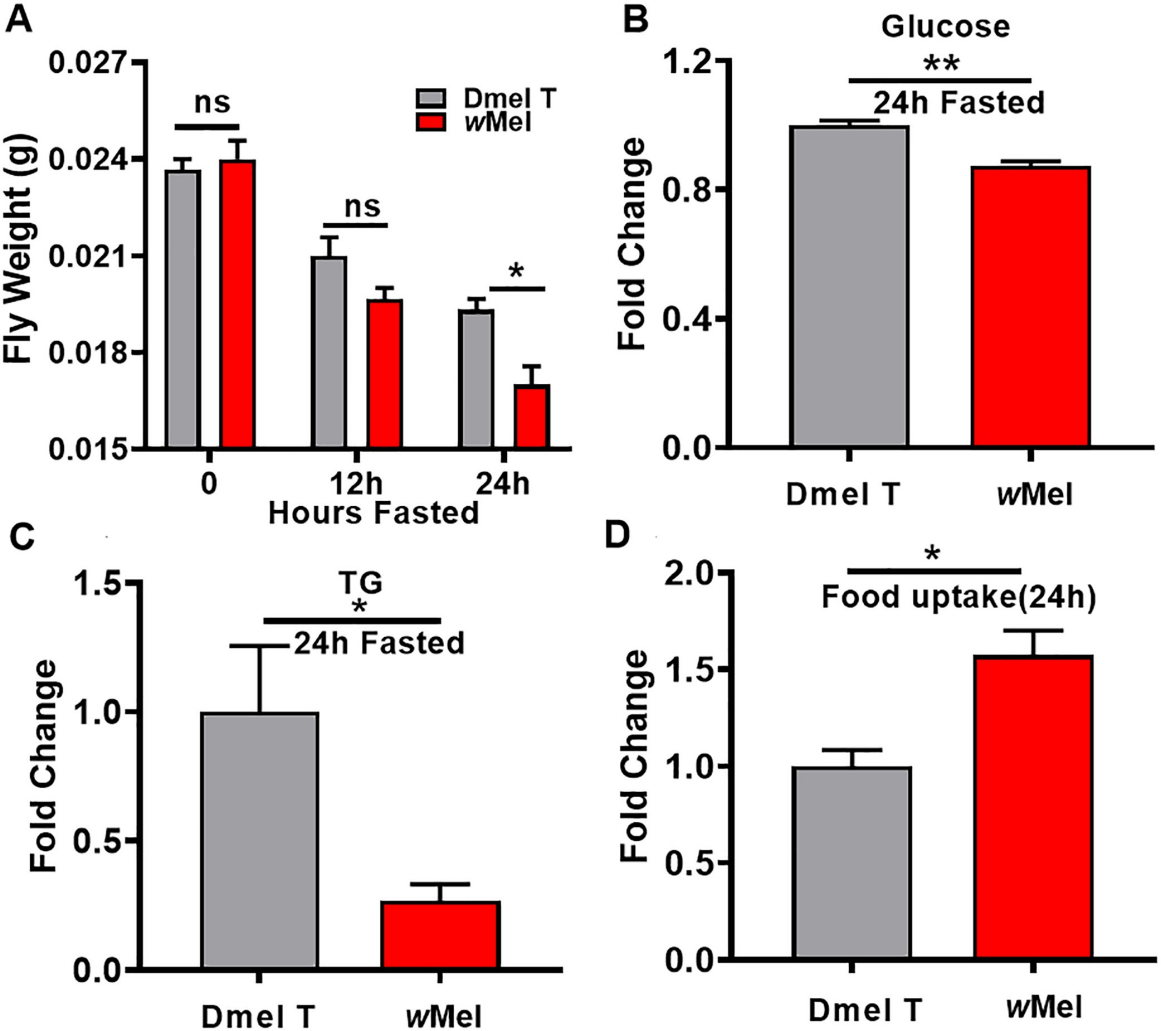
*Wolbachia* infection promoted the energy consumption and food intake in *D*. *melanogaster*. (A) The body weight of 30 male flies with fasting food and water at the three time points. (B) The fold change of D-glucose in Dmel T and *w*Mel flies after 24h fasted. (C) The fold change of triglycerides (TGs) in Dmel T and *w*Mel flies after 24h fasted. (D) The fold change of food uptake in Dmel T and *w*Mel flies during 24 h after eclosion. Dmel T: uninfected flies; *w*Mel: *Wolbachia*-infected flies. *P < 0.05; **P < 0.01. ns: not significant.

The above results have demonstrated that *Wolbachia* is able to enhance the energy metabolism in fly hosts. This infers that *Wolbachia* may speed up the energy expenditure in *D*. *melanogaster*. To verify this prediction, we compared the body weight of 30 male flies with fasting food and water for 12 h or 24 h between *Wolbachia* infected and uninfected males. The results revealed that after fasting food and water for 12 h, there was no apparent distinction in weight between these two *Wolbachia* status flies. However, *Wolbachia* infected flies fasting for 24 h exhibited significantly lighter body weight than the uninfected flies (Fig 7A). The levels of D-glucose and TGs were significantly reduced in *Wolbachia* infected flies relative to uninfected flies following 24h-fasting (Fig 7B and 7C). *Wolbachia* infection resulted in a significant increase in food uptake during 24 h after eclosion. The food uptake in *Wolbachia*-infected fly group was 1.58 times over that in uninfected groups (Fig 7D, *P*<0.05). These results indicate that *Wolbachia* infection accelerates energy expenditure in *D*. *melanogaster*.

### *Wolbachia* infection induces oxidative stress in *D. melanogaster*

**Fig 8:**
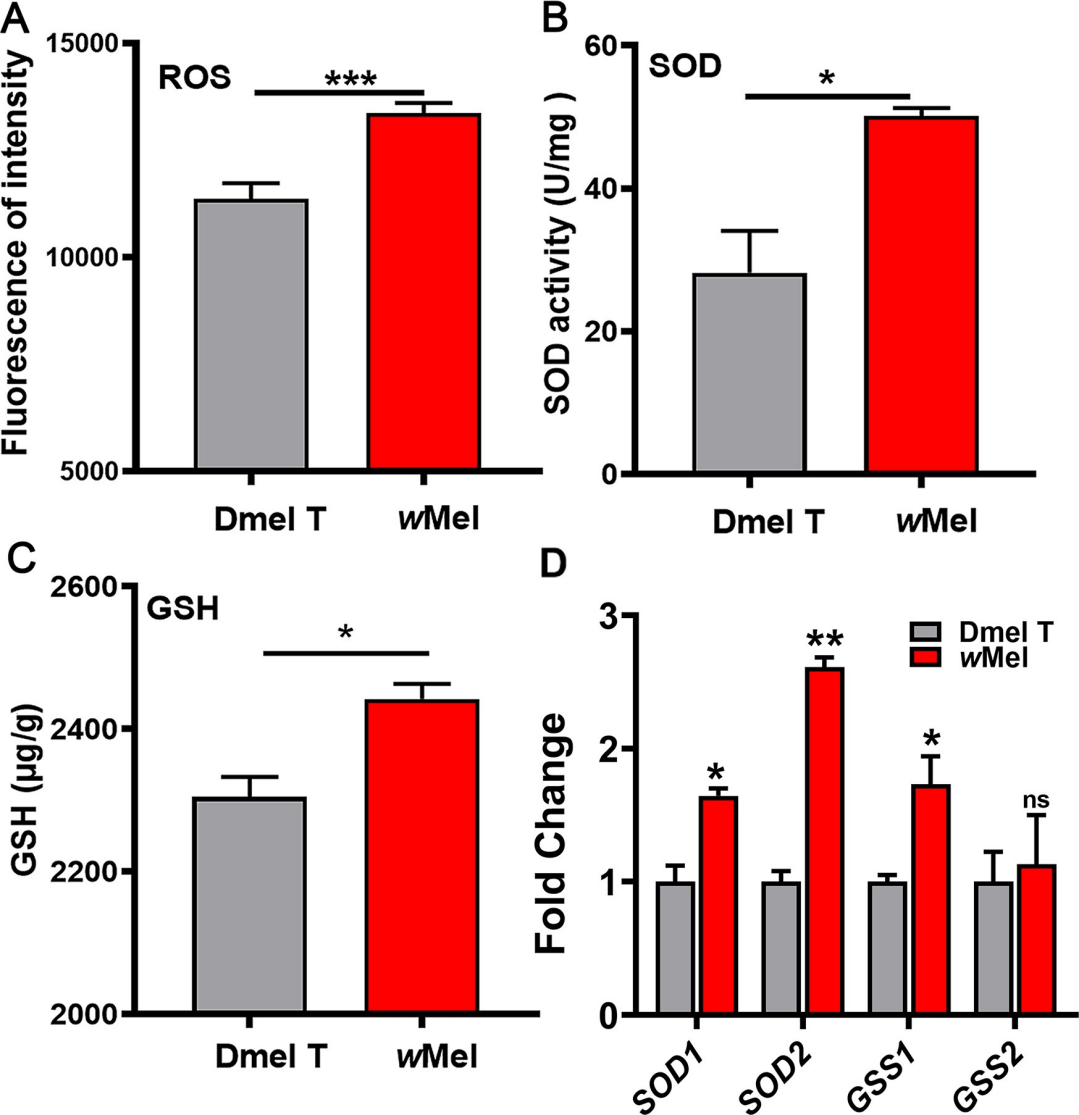
*Wolbachia* infection induces oxidative stress in *D*. *melanogaster*. *Wolbachia* induced the production of reactive oxygen species, ROS (A) and then increased superoxide dismutase, SOD (B) and glutathione, GSH (C). (D)*Wolbachia* infection caused changes in mRNA levels of SOD and GSH associated genes in 1d male flies. Dmel T: uninfected flies; *w*Mel: *Wolbachia*-infected flies. *P < 0.05; ***P < 0.001.

Since *Wolbachia* infection can stimulate the energy metabolism, thus it may also further cause oxidative stress because of excessive oxidation products. We therefore assayed the levels of reactive oxygen species (ROS), superoxide dismutase (SOD) and glutathione (GSH) in 1d males. We found that ROS, SOD, and GSH levels were all significantly increased in the 1d *Wolbachia*-infected males compared to uninfected ones (Fig 8A–8C). We further detected the transcription levels of SOD genes and GSH synthase genes in 1d *Wolbachia*-infected and uninfected male flies. The result showed that *SOD1* (coding for superoxide dismutase 1), *SOD2* (coding for superoxide dismutase 2), and *CSS1* (coding for glutathione synthetase 1) were all upregulated due to *Wolbachia* infection (Fig 8D). These results are consistent with an immune response against *Wolbachia* seen in other systems [39,40].

### *Wolbachia* infection damaged the formation of the mitochondrial nebenkern

**Fig 9:**
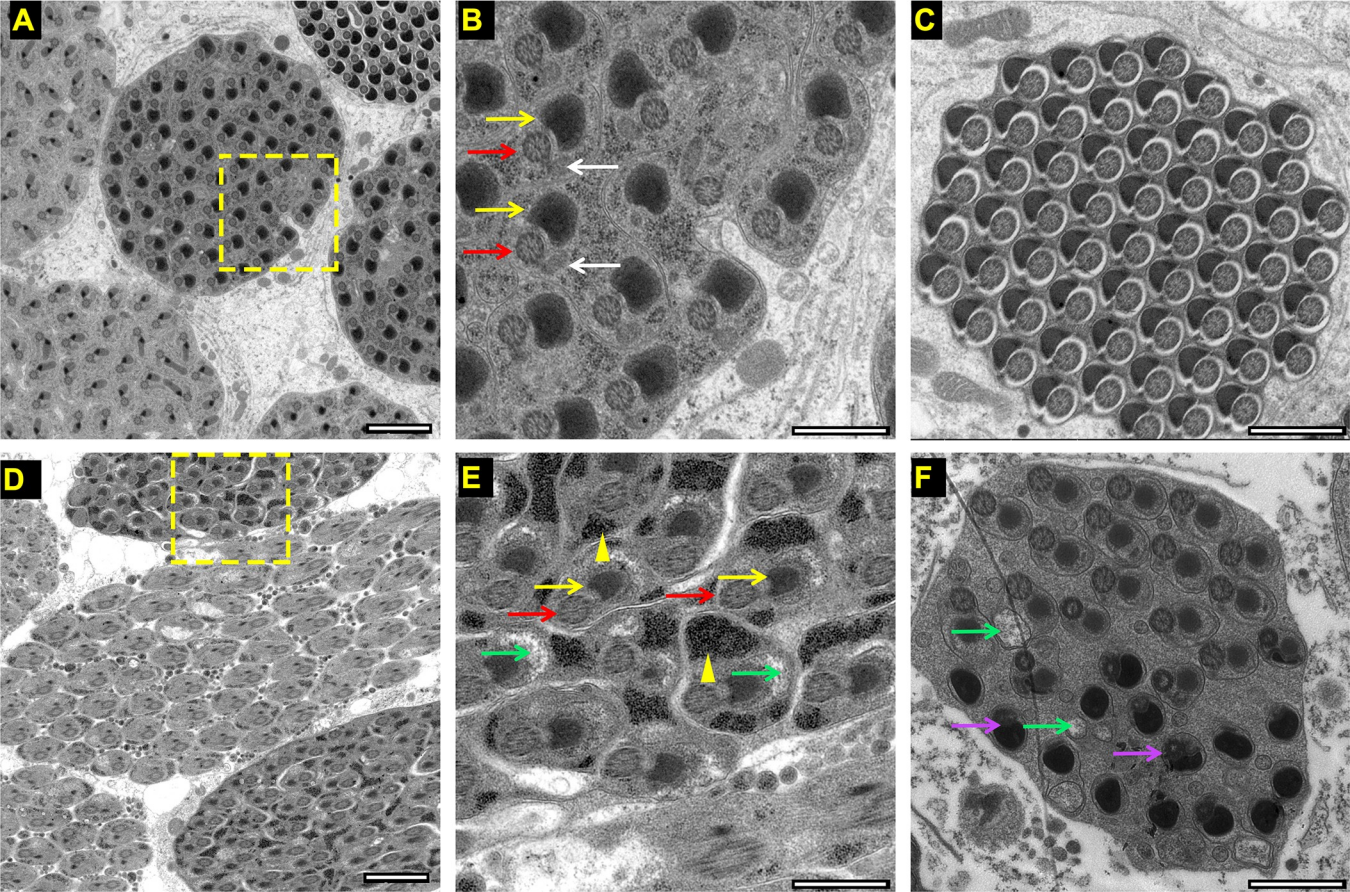
**Transmission electron microscope (TEM) of transverse section of testes of 1d *Wolbachia*-uninfected (A, B, C) and infected (D, E, F) *D*. *melanogaster*.** Yellow arrows indicate major mitochondrial derivatives. White arrows indicate minor mitochondrial derivatives. Red arrows represent axoneme. Yellow arrowheads show irregular black mass. Green arrows show vacuoles, and purple arrows indicate the fusing of the black mass with axoneme. Bars: 2 μm (A, D); 1 μm (B, C, E, F).

Normally, at the end of meiosis II, mitochondria aggregate and fuse to form two mitochondrial derivatives (major and minor) that wrap around each other in an onion-shaped structure called the nebenkern. The two mitochondrial derivatives elongate along the flagellar axoneme and provide structural rigidity for flagellar movement. By the end of spermatogenesis the minor one reduces its size and the major one accumulates paracrystalline material inside it[41]. Mitochondrial elongation has been shown to drive spermatid elongation [42]. Mutants for any genes involved in these processes cause male sterility [41,43]. Given *Wolbachia* infection induced oxidative stress in *D*. *melanogaster*, we speculated that *Wolbachia* infection may cause mitochondrial injury and mitochondria-originated nebenkern during late stage of spermatogenesis. The structural analyses of the axoneme and mitochondrial derivatives showed uniform distribution of spermatids containing one major mitochondrial derivative (yellow arrow) and one minor mitochondrial derivative (white arrow) associated with one axoneme (red arrow) in the cyst of uninfected fly testes (Fig 9A and 9B). The paracrystalline material accumulated in the major mitochondrial derivative (yellow arrow in Fig 9B) as described previously[43]. At the end of spermatogenesis, sperms were highly oriented in a cyst. The major mitochondrial derivatives condensed to form a black half circle nebenkern coupled with an axoneme while the minor one regressed (Fig 9C). However, the spermatid frequently contained only one typical major mitochondrial derivative (yellow arrows in Fig 9E) next to one axoneme in the cyst of *Wolbachia*-infected fly testes. Furthermore, many black masses with different sizes (yellow arrowhead in Fig 9D and 9E) seemed to be the accumulated peroxides were observed in the spermatid. Some vacuoles appeared in most of cysts with partial vacuoles being unnaturally large (green arrows Figs 9D–9F and S6), which may be associated with the abnormal mitochondrial derivatives. Notably, most of the sperm cells could form the flagellar structure such as axoneme and nebenkern (S6 Fig). In addition, the black mass of some special abnormal cysts (6.67%, n = 30) was observed to be condensed and fused with the axoneme (purple arrows in Fig 9F).

## Discussion

**Fig 10:**
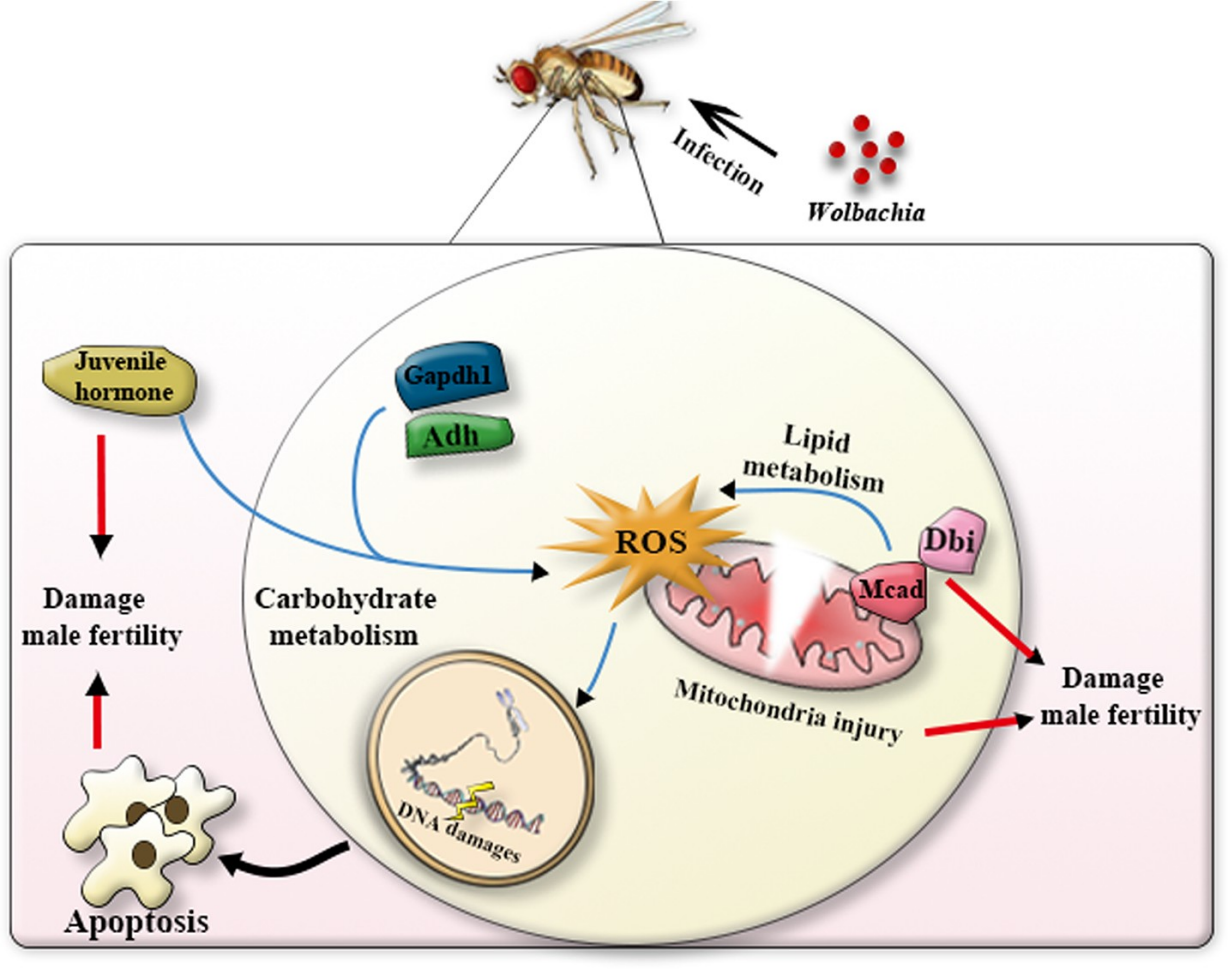
A simple model showing that *Wolbachia*-induced energy output in 1d males affects male fertility in *D*. *melanogaster*.

It is well known that *Wolbachia* infection can manipulate the reproduction of their insect hosts. However, the underlying metabolic mechanisms by which the endosymbionts resulting in paternal defects in their insect hosts are yet to be established. Previous studies have suggested that carbohydrate and fatty acids metabolism are highly associated with spermatogenesis[23,24,44]. The objective of the current study was to investigate the mechanisms of paternal defects from the aspect of metabolic alterations induced by *Wolbachia*. We examined the metabolic profiling in both *Wolbachia*-bearing and *Wolbachia*-free male *D*. *melanogaster* using untargeted and targeted metabolomics approaches. Differential metabolites and their located metabolic pathways were figured out. Gene expression and fertility analyses were also employed to elucidate the relevance of metabolism and male reproduction. The results indicated that *Wolbachia* infection significantly up-regulated carbohydrate and lipid metabolism, and cofactors and vitamins metabolism of *D*. *melanogaster*. Nevertheless, over-activation of energy metabolism is usually accompanied by production of more ROS and hyperoxide, which damaged male fertility (Fig 10).

As an intracellular endosymbiont, *w*Mel *Wolbachia* lost many genes, including many of the metabolism-related genes [45]. Therefore, they must rely on, to a large extent, the host to obtain enough components for their own survival and proliferation. In this study, we found that *w*Mel *Wolbachia* infection stimulated food uptake and carbohydrate metabolism of the host *D*. *melanogaster*, manifested by elevated levels of glucose 6-phosphate, fructose 6-phosphate, and mannose 6-phosphate. The level of glucose in *Wolbachia*-bearing male flies is significantly higher than that in *Wolbachia*-free males. Consistently, the transcription levels of *Gapdh* and *Adh* relating to glycolysis appeared to be significantly up-regulated due to *Wolbachia* infection. On the contrary, *Wolbachia* infection induced significant depletion of trehalose and glycogen levels of *D*. *melanogaster*. These suggest that *Wolbachia* infection promotes the catabolism of trehalose and glycogen to glucose and thus enhancing the glycolysis. These results were well consistent with previous studies in whole adult female mosquitos and *D*. *melanogaster* testes[19,20,46]. It was noteworthy that *Wolbachia* infection significantly elevated in the levels of (10S)-juvenile hormone III acid diol and (10S)-juvenile hormone III diol (S2 Table), two important metabolites involved in the biosynthetic pathway of the juvenile hormone (JH). This is in accord with our previous observations in the 3^rd^ instar larval testes and adult testes with enhanced JH signalling in male fly testes[29]. Previous evidence showed that carbohydrate metabolism is involved in JH signal pathway. When JH synthesis is blocked, the proportion of glucose to trehalose is significantly reduced due to the reduced of trehalase decomposition without its biosynthesis affection. Furthermore, artificial supplementation with JH analogs can restore the balance of the carbohydrates[47,48]. The accumulation of JH may be related to the change in carbohydrate metabolism and the spermatogenesis directly caused by *Wolbachia* infection. Over-activation of the JH signalling pathway in male *D*. *melanogaster* was also verified to cause severe declining male fertility[29].

Of particular note was that *Wolbachia* infection significantly enhanced lipid metabolism in *D*. *melanogaster*, in which the levels of linolenoyl-CoA, palmitoleic acid, and gamolenic acid involved in lipid metabolic pathway were markedly elevated. *Wolbachia*-infected male flies exhibited higher levels of TG, as well as many other intermediate products of fatty acid metabolism, such as C14, C16 and C18, than those in uninfected flies. Consistently, *Wolbachia* infection significantly up-regulated the mRNA levels of *Dbi* and *Mcad* encoding acyl-CoA binding protein 2 and acyl-CoA dehydrogenase involved in fatty acid beta-oxidation, respectively[38,49,50]. *Wolbachia* also lack key biosynthesis genes for lipid metabolism and have to heavily utilise host lipids to serve their own propagation[45,46,51,52]. Actually, many genes encoding enzymes related to fatty acid synthesis were previously found to be significantly upregulated in the 3^rd^ instar larval testes by *Wolbachia* infection[19]. A 2.36-fold increase in fatty acid synthase (*Fas*) mRNA expression in *w*Mel-infected *A*. *aegypti* mosquitoes was also observed before[53]. Furthermore, overexpression of *Dbi* or *Mcad* induced a phenotype similar to the paternal defects induced by *Wolbachia*, that is, the hatch rate of the eggs significantly decreased when the females mated with either *Dbi* or *Mcad* overexpressing males. Collectively, these suggest that *Wolbachia* infection enhances the fatty acid metabolism by up-regulating expressions of some genes related to fatty acid metabolism, thus impairing male fertility in *Drosophila* host.

The enhancement of energy uptake and carbohydrate and lipid metabolism of *D*. *melanogaster* by *Wolbachia* infection may make the host environment more favourable for *Wolbachia* survival and propagation. However, excessive activation of energy metabolism is usually accompanied by redox condition. Many oxidative phosphorylation genes in *Wolbachia* infected flies were up-regulated[19,20]. Here some important vitamins and coenzymes were significantly up-regulated in flies infected with *Wolbachia*; such as nicotinic acid (B3), pantothenic acid (B5) and vitamin H (B7), among which pantothenic acid showed significant up-regulation in both modes (S2 Table). Interestingly, all of those up regulated vitamins belong to the vitamin B family. *Wolbachia* has been shown to have highly conserved pathways for synthesis of vitamins [54] and can also provide vitamins, biotin, and riboflavin to their hosts[55–57]. Nicotinic acid (B3) is generally present in the form of nicotinic acid amide co-enzyme (NAD, nicotinamide-adenine dinucleotide and NADP, nicotinamide-adenine dinucleotide phosphate)[58], pantothenic acid is an obligate precursor of coenzyme A (CoA)[59,60], and Vitamin H is the coenzyme of acetylcoa carboxylase, pyruvate fusinase and many other enzymes[61], all of them play vital roles in the oxidation-reduction reaction. Therefore, *Wolbachia* infection may induce paternal defects in hosts through changing the vitamin levels and thus the oxidation-reduction pathway.

Excess oxidative phosphorylation further induces oxidative stress and impairs the function of mitochondria. In the current study, *Wolbachia* infection markedly elevated levels of ROS, SOD and GSH of *D*. *melanogaster*, which were consistent with previous studies on whole host adults or host testes[35,40,62–64]. It is known that oxidative stress can cause DNA damage in spermatocytes and reduce the number of spermatocytes, ultimately resulting in apoptosis of spermatocytes[35,63,65,66]. This notion was further supported by the observation of *Wolbachia* infection-induced deficiency of mitochondria-derived nebenkern development during spermatogenesis in *D*. *melanogaster* (Fig 9). These results are consistent with the TA model for explaining CI, in which *Wolbachia* may deposit some toxic material in maturing sperm, which can cause paternal defects in embryonic development[17]. Notably, TA model also showed that the antidote CifA have three putative domains, one of which is a catalase-related domain involved in the degradation of ROS[4] in rescuing the modification in sperm.

In conclusion, for survival and proliferation, *Wolbachia* need to promote host metabolism in a variety of metabolic pathways including carbohydrate and lipid metabolism. However, *Wolbachia* infection-induced enhancement of energy metabolism, oxidative stress and impaired mitochondrial function of *D*. *melanogaster* may cause defects in the development of mitochondria-derived nebenkern during spermatogenesis, ultimately leading to paternal reproductive defects.

## Supporting information

S1 Table Primers used in this study. (DOCX)

S2 Table Potential differential metabolites induced by *Wolbachia* in *D*. *melanogaster*. (XLSX)

S3 Table Overexpression of *Dbi* or *Mcad* ubiquitously significantly decreased male fertility of *D*. *melanogaster*. (DOCX)

S1 Fig *Drosophila* genetics hybridization patterns. Crosses between *Wolbachia*-free balance and *UAS-Dbi*(or *UAS-Mcad*) flies to generate a Co-overexpression hybrid line(A). A cross between *Wolbachia-*infected balance and bamGal4 flies to generate the *Wolbachia*-infected bamGal4 line(B). I+: *Wolbachia*-infected.
(TIF)

S2 Fig Absolute quantification of *rp49* in 1-day *Wolbachia-*infected or *Wolbachia*-free males and 1day testes of males. (TIF)

S3 Fig Annotations of potential metabolites in the map of metabolic pathways (Kegg ID: ko01100) in positive ion mode (ESI+) (A) and negative ion mode (ESI-) (B). (TIF)

S4 Fig Mass spectrogram fatty of acid methyl ester (FAME)-based GC-MS analysis of long chain fatty acids (A) and short chain fatty acids (SCFA)-based GC-MS analysis of short chain fatty acids (B) levels in 1d males. (TIF)

S5 Fig QRT-PCR analyses demonstrated the *Dbi* (A, C, E, G) and *Mcad* (B, D, F, H) expression levels relative to the corresponding controls in males used for fertility test. *P<0.05; **P<0.01; I-: *Wolbachia*-free; I+: *Wolbachia*-infected.
(TIF)

S6 Fig Transverse section of testes of 1d *Wolbachia-*infected *D*. *melanogaste*r. Some cysts contain sperms with normal morphology (A), but some other cysts contain large or small vacuoles and deformed sperms although the sperms can form the flagellar structure such as axoneme and nebenkern (B, C. D). Bars: 4 μm.
(TIF)
