# Supplementary material for: Metabolomics provide new insights into mechanisms of Wolbachia-induced paternal defects in Drosophila melanogaster
Source: PLoS Pathog. 2021 Aug 12;17(8):e1009859. doi: 10.1371/journal.ppat.1009859 (PMC8384202; doi:10.1371/journal.ppat.1009859)
Supplement: S1 Table — (DOCX) [file ppat.1009859.s001.docx]

**S1 Table.** Primers used in this study

| **Transcript** | **Forward primer (5’-3’)** | **Reverse primer (5’-3’)** |
| --- | --- | --- |
| *wsp* | TGGTCCAATAAGTGATGAAGAAAC | AAAAATTAAACGCTACTCCA |
| *Gapdh 1* | TGGATCTTACCGTCCGCTTG | ACCACCTCCTCATCGGTGTA |
| *Adh* | GACGGCCATTCTGGACTTCT | TAGACGGGCACCTGGTAGAT |
| *CG6262* | CACTTTGTGGACATGAGCTGC | GGATCATTGAAGTGCTTCTCGAC |
| *Gyg* | GTCGGAAGAAACTGGTAGCTCA | TAGGGTGGAGAAGGGGTGG |
| *Dbi* | TCTGTGGCAAACAACACACAAC | AGGAACTCGTCATCACTGGG |
| *Mcad* | GAAAAACACATCGCTGGGACA | TTCGAAACGGAACCCACTCG |
| *SOD1* | ACACGAGCTGAGCAAGTCAA | CAGTGGCCGACATCGGAATA |
| *SOD2* | ATAAGCATCTGCGGATCGGA | ACGCCTTTGTGAAGCGTTATC |
| *GSS1* | ACGAGGAGTTCATCACGACG | ACTCTAGGCAACAATCTGGGTG |
| *GSS2* | CAAAGCTCTCAACCGCCAAC | GGTCTAGAAAGCTTCTACTTCTGC |
| *rp49* | CGGTTACGGATCGAACAAGC | CTTGCGCTTCTTGGAGGAGA |
