# Supplementary material for: Metabolomics provide new insights into mechanisms of Wolbachia-induced paternal defects in Drosophila melanogaster
Source: PLoS Pathog. 2021 Aug 12;17(8):e1009859. doi: 10.1371/journal.ppat.1009859 (PMC8384202; doi:10.1371/journal.ppat.1009859)
Supplement: S3 Table — (DOCX) [file ppat.1009859.s003.docx]

**S3 Table.** Overexpression of *Dbi* or *Mcad* ubiquitously significantly decreased male fertility of *D. melanogaster*

| Crossgroup | Cross flies（♂ × ♀） | Egg hatch (%) | Egg  counted | Comparison |
| --- | --- | --- | --- | --- |
| 1 | actGal4(I-)>*w^-^* × Dmel T | 80.81 ± 0.26 | 1262 |  |
| 2 | actGal4 (I-) >*UAS-Dbi*;*UAS-Dbi* × Dmel T | 51.94 ± 4.38 | 1197 | 2 *vs*. 1  *P*<0.05 |
| 3 | actGal4(I-)>*UAS-Mcad* × Dmel T | 42.16 ± 3.12 | 423 | 3 *vs*. 1  *P*<0.05 |

I-: *Wolbachia*-free; Dmel T: tetracycline treated (*Wolbachia*-free) *Drosophila melanogaster*;
