# Supplementary figures and images for: Metabolomics provide new insights into mechanisms of Wolbachia-induced paternal defects in Drosophila melanogaster
Source: PLoS Pathog. 2021 Aug 12;17(8):e1009859. doi: 10.1371/journal.ppat.1009859 (PMC8384202; doi:10.1371/journal.ppat.1009859)

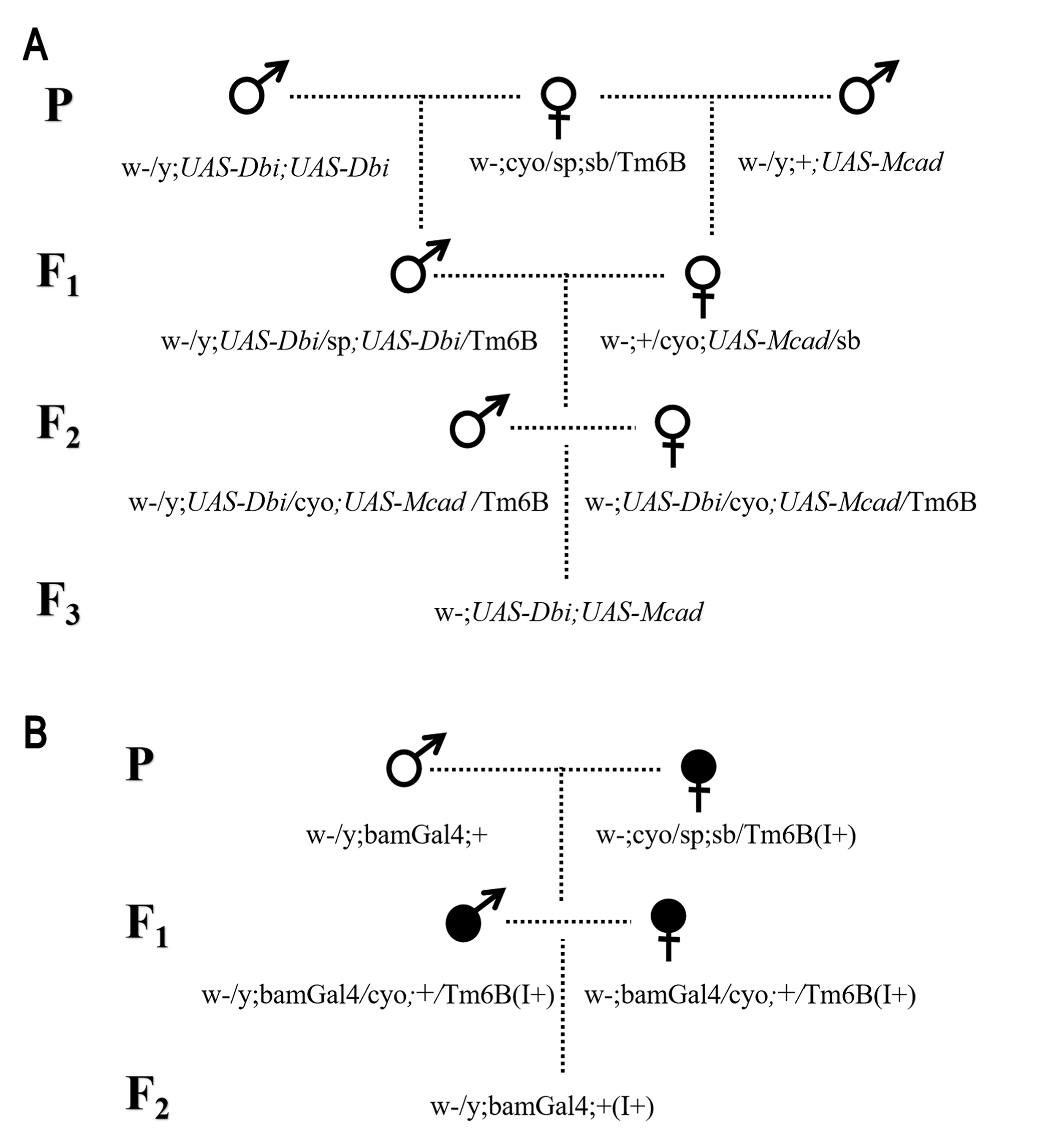

Supplement: S1 Fig — Crosses between Wolbachia-free balance and UAS-Dbi(or UAS-Mcad) flies to generate a Co-overexpression hybrid line(A). A cross between Wolbachia-infected balance and bamGal4 flies to generate the Wolbachia-infected bamGal4 line(B). I+: Wolbachia-infected. (TIF) [file ppat.1009859.s004.tif]

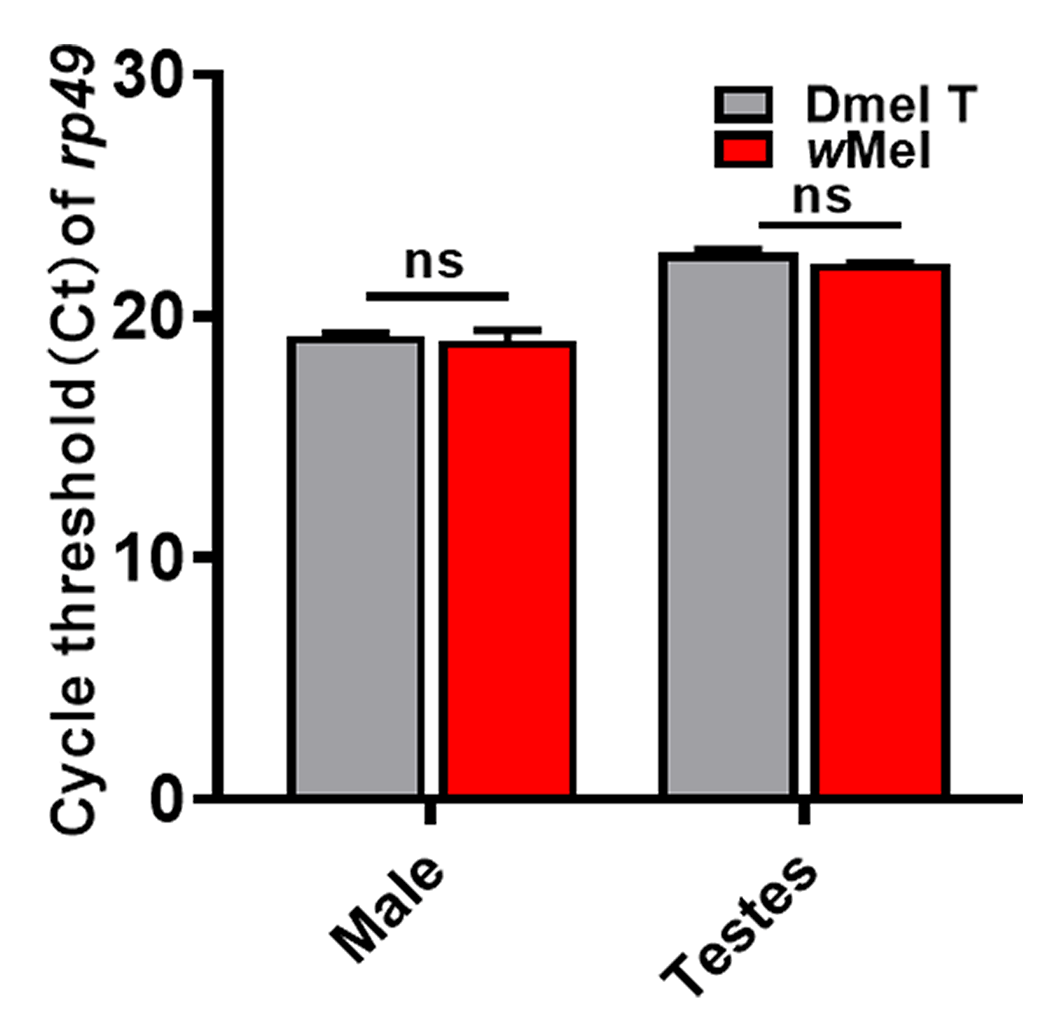

Supplement: S2 Fig — (TIF) [file ppat.1009859.s005.tif]

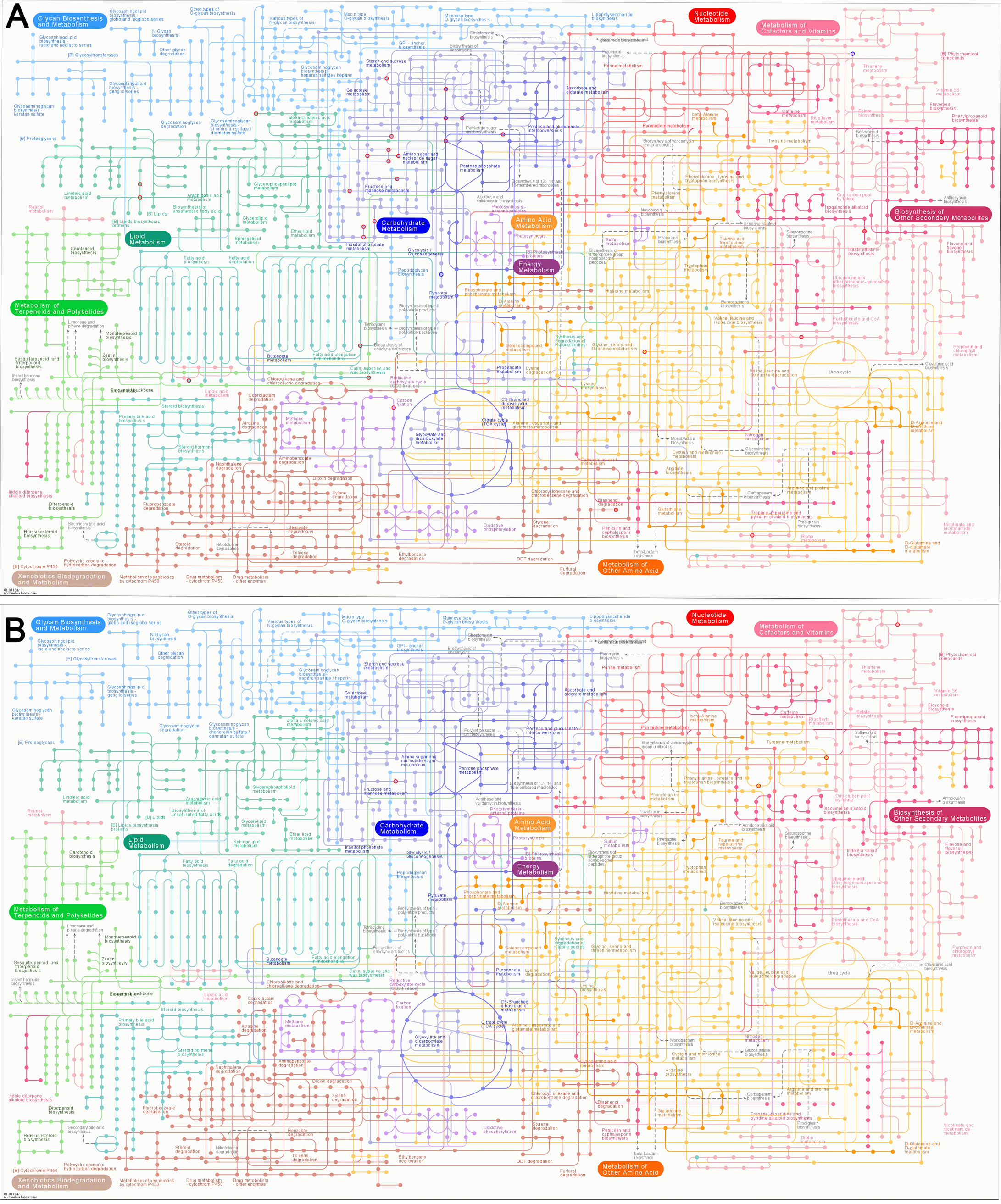

Supplement: S3 Fig — (TIF) [file ppat.1009859.s006.tif]

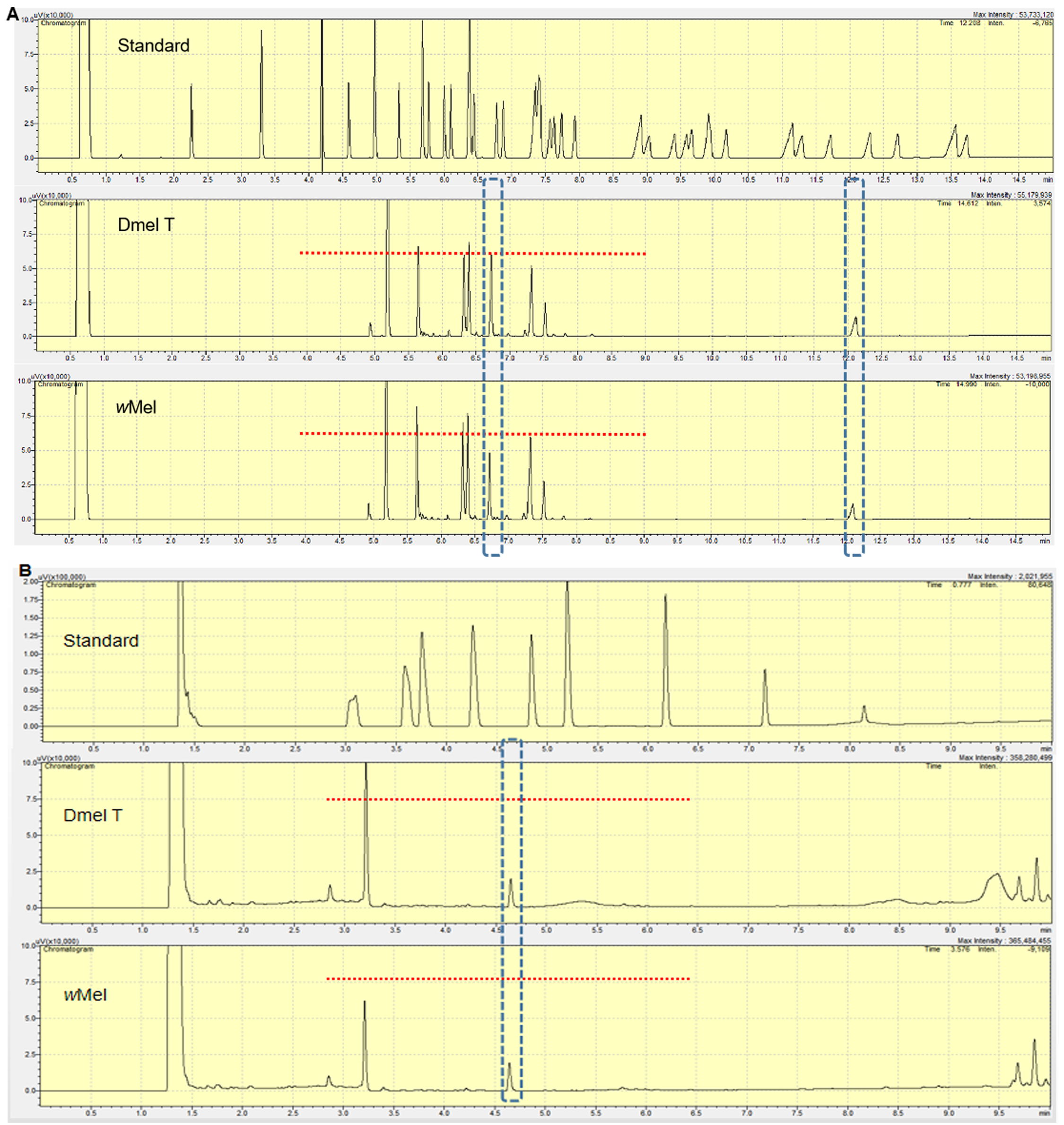

Supplement: S4 Fig — (TIF) [file ppat.1009859.s007.tif]

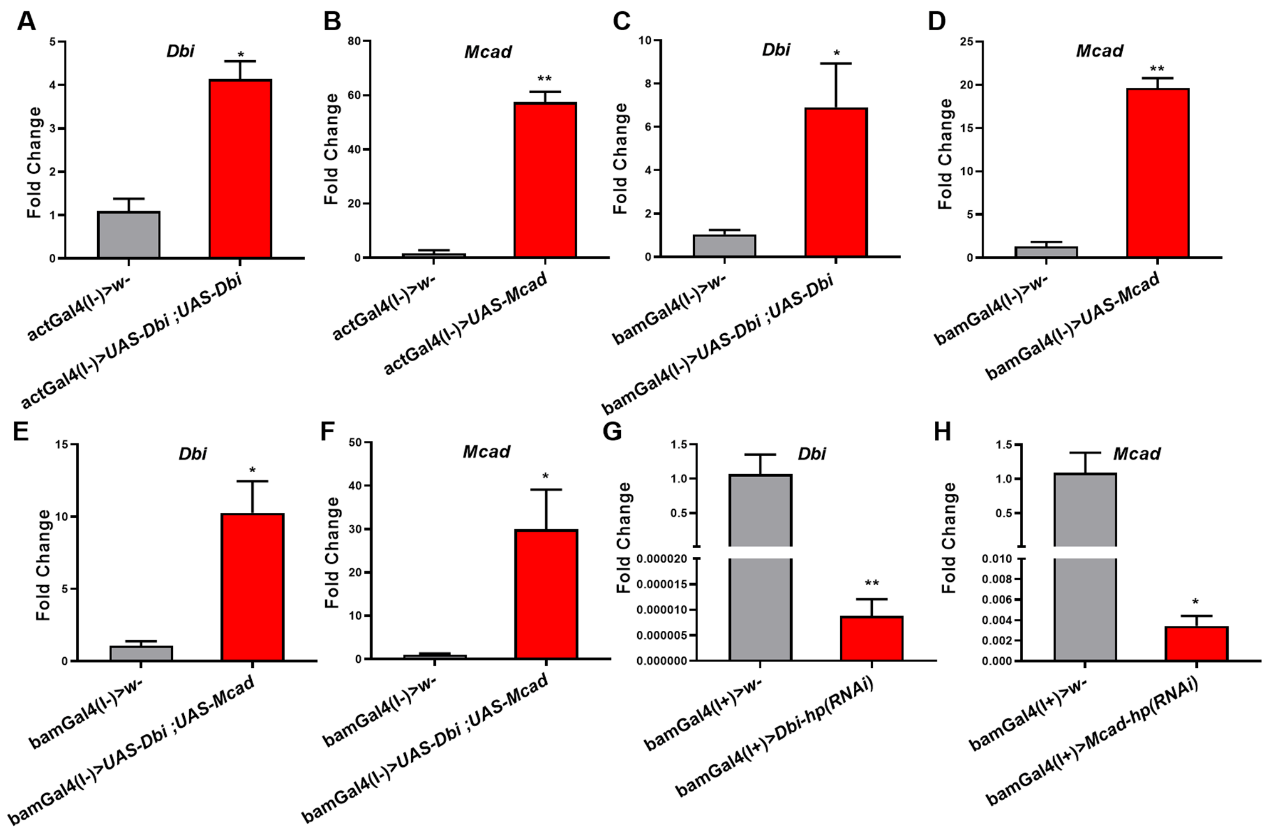

Supplement: S5 Fig — *P<0.05; **P<0.01; I-: Wolbachia-free; I+: Wolbachia-infected. (TIF) [file ppat.1009859.s008.tif]

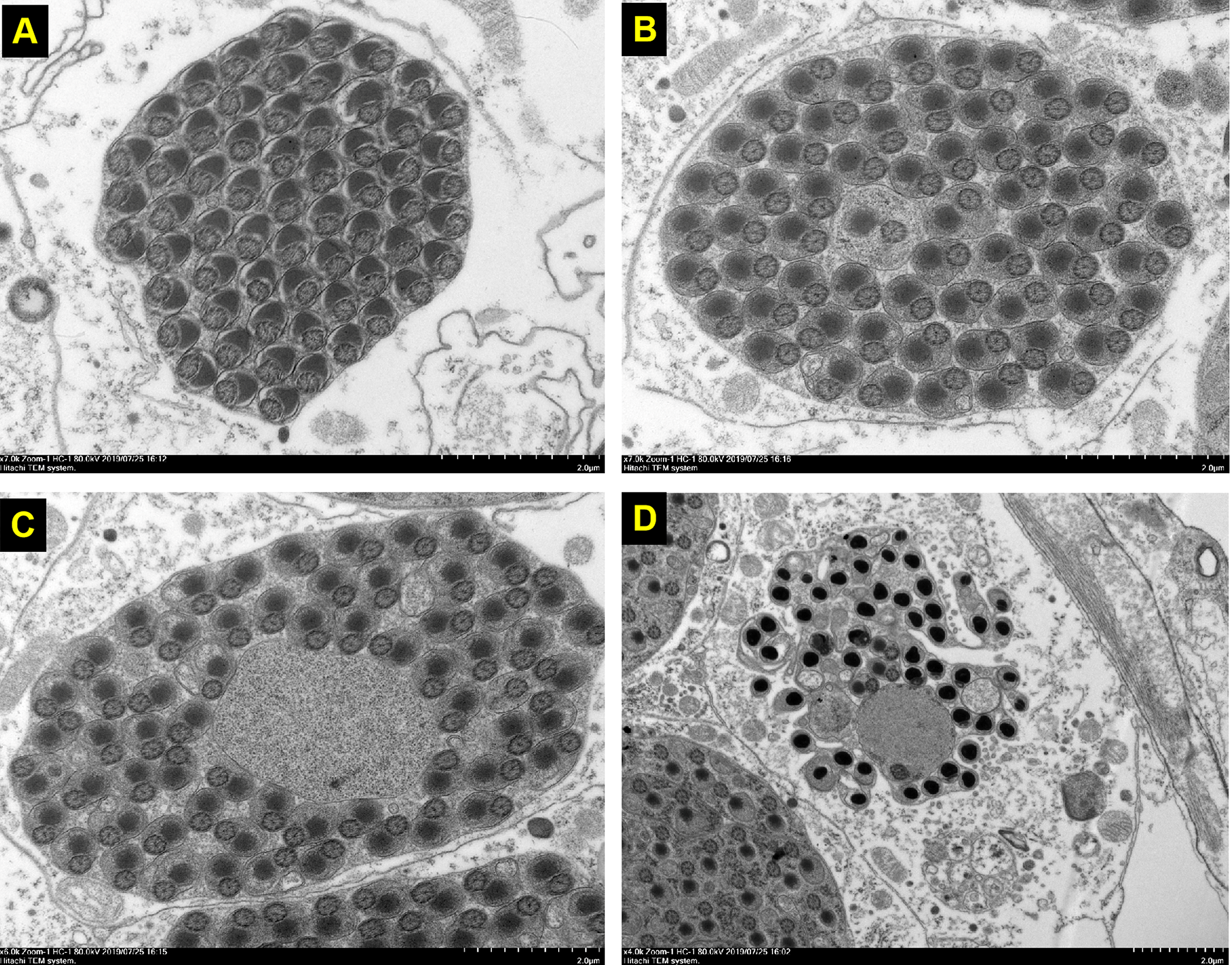

Supplement: S6 Fig — Some cysts contain sperms with normal morphology (A), but some other cysts contain large or small vacuoles and deformed sperms although the sperms can form the flagellar structure such as axoneme and nebenkern (B, C. D). Bars: 4 μm. (TIF) [file ppat.1009859.s009.tif]
